# Adaptive selection drives TRPP3 loss-of-function in an Ethiopian population

**DOI:** 10.1038/s41598-020-78081-z

**Published:** 2020-12-02

**Authors:** Sandra Walsh, Mercè Izquierdo-Serra, Sandra Acosta, Albert Edo, María Lloret, Roser Moret, Elena Bosch, Baldo Oliva, Jaume Bertranpetit, José Manuel Fernández-Fernández

**Affiliations:** 1 Institut de Biologia Evolutiva (UPF-CSIC), Universitat Pompeu Fabra, Dr. Aiguader, 88, 08003 Barcelona, Catalonia Spain; 2 Laboratory of Molecular Physiology, Department of Experimental and Health Sciences, Universitat Pompeu Fabra, 08003 Barcelona, Spain; 3 Centro de Investigación Biomédica en Red de Salud Mental (CIBERSAM), 43206 Reus, Spain; 4 Structural Bioinformatics Lab, Department of Experimental and Health Sciences, Universitat Pompeu Fabra, 08003 Barcelona, Spain

**Keywords:** Biophysics, Evolution, Genetics, Molecular biology, Physiology, Structural biology

## Abstract

TRPP3 (also called PKD2L1) is a nonselective, cation-permeable channel activated by multiple stimuli, including extracellular pH changes. TRPP3 had been considered a candidate for sour sensor in humans, due to its high expression in a subset of tongue receptor cells detecting sour, along with its membership to the TRP channel family known to function as sensory receptors. Here, we describe the functional consequences of two non-synonymous genetic variants (R278Q and R378W) found to be under strong positive selection in an Ethiopian population, the Gumuz. Electrophysiological studies and 3D modelling reveal TRPP3 loss-of-functions produced by both substitutions. R278Q impairs TRPP3 activation after alkalinisation by mislocation of H^+^ binding residues at the extracellular polycystin mucolipin domain. R378W dramatically reduces channel activity by altering conformation of the voltage sensor domain and hampering channel transition from closed to open state. Sour sensitivity tests in R278Q/R378W carriers argue against both any involvement of TRPP3 in sour detection and the role of such physiological process in the reported evolutionary positive selection past event.

## Introduction

The action of positive (also called adaptive) selection can be identified in our genome by exploring the genome-wide landscape of the footprints of the so-called classical sweeps^1^. Recognizing where in the genome adaptation has happened is a fully blind process in relation to the information of the genome, and many methods have been described to have full genome scans of adaptive selection (see^2^ for a review). However, the biological interpretation of such footprints is not direct. Indeed, besides identifying where in the genome the sweep occurred, the obvious next step is to recognize the genetic bases of the selected phenotype. The understanding of the relationship between the selection signal and the selected trait is, in general, much more complex, with many cases not solved. So far, the most powerful and interesting method to associate phenotypic adaptations is population specific adaptation^3–5^. There are some well-known cases for which the adaptation has been fully interpreted, from the detection of the signal to the specific genetic change and into the adaptive phenotype, like the lactase persistence^6,7^, the adaptation to high altitudes^8–11^ and diet^12^. In the recent years, the explosion of the production of genomic data has allowed to undertake whole genome scans of positive selection for several human populations. So far, the most reliable functional adaptive selection are associated to protein-coding genes, but for many cases, the detection of a selection signal is far from being functionally interpreted, being the main reason the lack of precision of the selection footprints, which usually comprise several kilobases with many different (and often pleiotropic) genes and several functional genetic variants, many still not well known or annotated in the genome.

As in the case of the identification of adaptive selection in fat metabolism in Inuit^12^ and lactase persistence mainly in Europe^6,7^, food resources are main drivers of adaptation. Therefore natural selection may have played an important role in shaping the genetic variation of the human taste system. Food resources and potential dangers vary across different environments and thus the human taste system has potentially evolved in a population specific way. These potential adaptations are likely to be very population specific as a survey on selection in the main taste-related genes in three divergent human populations (Yoruba from Africa, Han from China and Central Europeans) did not find main adaptive differences in taste, while specific adaptations were found for biotransformation^13^.

The mammalian tongue contains gustatory receptors regulating basic taste types, providing an evolutionary old hedonic compass for what to ingest and what not to ingest^14^. One of the main hypotheses of the evolutionary sour tasting function is that it could warn against the acidic ingestion of rotten, unripe or fermented food^15^. In the recent years, transient receptor potential (TRP) and particularly TRPP3 had been considered a candidate for acid receptor since the ablation of taste receptor cells expressing TRPP3 in mice deplete the acid-induced responses in gustatory nerve recordings^16,17^. TRPP3 (Transient Receptor Potential Polycystin-3, also called Polycystic Kidney Disease 2-Like 1 (PKD2L1) or polycystin-L) belongs to the TRPP subfamily of ion channels that are characterized by large extracellular domains^18,19^. Human TRPP3 forms a nonselective, cation-permeable channel in a tetrameric structure. Each TRPP3 monomer has a voltage sensor domain (VSD) formed by the S1 to S4 transmembrane α-helices, a pore domain formed by the S5 to S6 helices, and a polycystin mucolipin domain (PMD) in the extracellular loop between transmembrane segments S1 and S2, which rests on top of the VSD of the same monomer and the adjacent pore domain of the tetrameric channel^19^. TRPP3 responds to multiple stimuli that include voltage membrane changes, mechanical stress, hypo-osmotic shock, temperature changes and alterations in extracellular pH^20–23^. Hence, TRPP3 is co-expressed and interacts with the Polycystic Kidney Disease 1-Like 3 (PKD1L3) protein in a subset of receptor cells at the tongue taste buds of mammals^24^, where the TRPP3/PKD1L3 channel complex was suggested to partly contribute to sour taste responses due to its activation after washout of an acid stimulus (acid-evoked off-response)^25–28^. Accordingly, in humans, two patients with sour taste ageusia have been reported and neither had detectable TRPP3 transcripts^29^. However, whereas it is well accepted that ablation of taste receptor cells expressing TRPP3 eliminates sour taste responses^30^, receptors other than TRPP3 and PKDL13 proteins have been proposed to be the main responsible for acid/sour detection. Indeed, genetic or functional knockout of TRPP3 and/or PKD1L3 in mice only produces a mild reduction (by 25–45%) in acid taste responses^27^ or has no effect at all^31^. Interestingly, Otopetrin1 (OTOP1) has been identified in TRPP3-expressing, acid-detecting taste receptor cells of mice as the ion channel required for conduction of H^+^ currents in response to extracellular acidification^32^. There, OTOP1 is essential for sour sensing in the taste system^33,34^. OTOP1 is evolutionarily conserved from nematodes to humans^32,35,36^, and human OTOP1 also works as an H^+^ channel of similar properties to murine OTOP1^32^. Overall, these results point out to OTOP1 as the main sour receptor in humans.

In this study we depart from a signal of selection in the genome, recognize the gene *TRPP3* and two non-synonymous variants as the selection target, and use molecular physiology techniques measuring channel activity and 3D molecular models to characterize the functional alterations produced by the two putative adaptive substitutions. Finally, in order to establish a link between the adaptive genotype and a potential adaptive external phenotype we look for differences in sour taste recognition (as the minimum citric acid concentration where a subject can recognize sour taste). Our results show a total decrease of the double mutant channel activity as seen in the electrophysiological experiments, which is in agreement with the 3D structure modelling of the two substitutions. On the phenotype, our results give further support to previous doubts on the involvement of TRPP3 in sour taste recognition.

## Results

### Identification of adaptive selection in the TRPP3 gene in the Gumuz population

**Figure 1:**
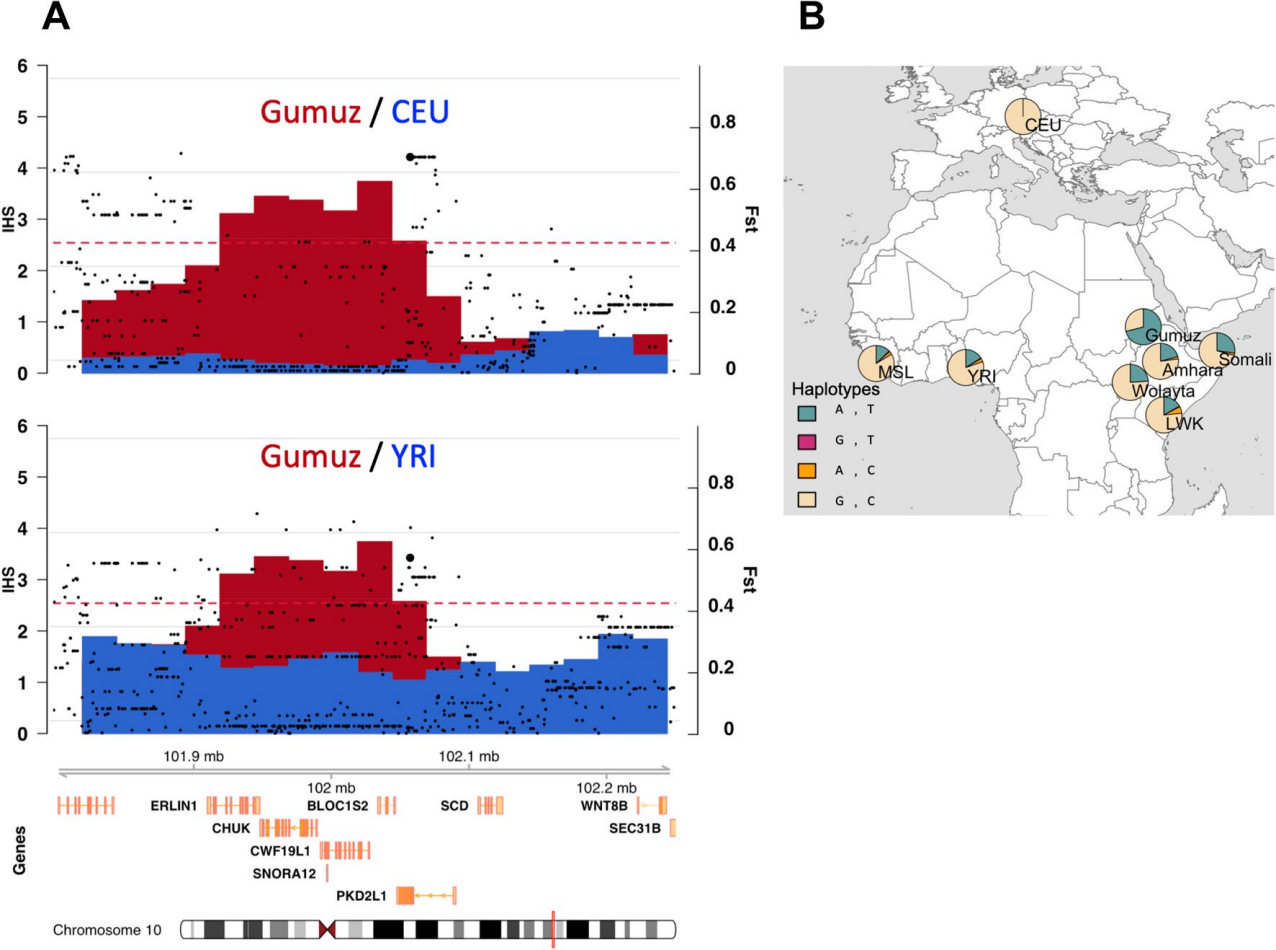
Positive selection signal in the *TRPP3* region of the Gumuz population. (**A**) Positive selection analysis (iHS and Fst) of the Gumuz population compared to CEU (upper panel) and YRI (lower panel) populations. Blue and red bars represent the absolute mean iHS of a 30 kilobase (kb) window (left y-axis). Red dotted lines indicate the 99.99th percentile thresholds of the absolute mean iHS per 30 kb window reported in Walsh et al., in press that were obtained from extensive population genetics neutral simulations. Black dots correspond to the Fst values per site (as a measure of population differentiation) between the Gumuz and CEU (upper panel) and YRI (lower panel) population; the right y-axis indicates the scale of Fst values. Variants rs17112895 and rs7909153 are indicated in bigger black dots. (**B**) Spatial distribution of haplotypes per population. Pie plots indicate the population frequencies of each combination of haplotypes containing both substitutions in the Gumuz, Amhara, Somali, Wolayta from Ethiopia, YRI: Yoruba from Nigeria, LWK: Luhya from Kenya, MSL: Mende from Sierra Leone, CEU: Europeans. Map and pie plots were generated with R (v3.5.3)^72^ using the packages rworldmap (v1.3-6, http://journal.r-project.org/archive/2011-1/RJournal_2011-1_South.pdf), rworldxtra (v1.01, https://CRAN.R-project.org/package=rworldxtra) and mapplots (v1.5.1, https://CRAN.R-project.org/package=mapplots).

**Figure 2:**
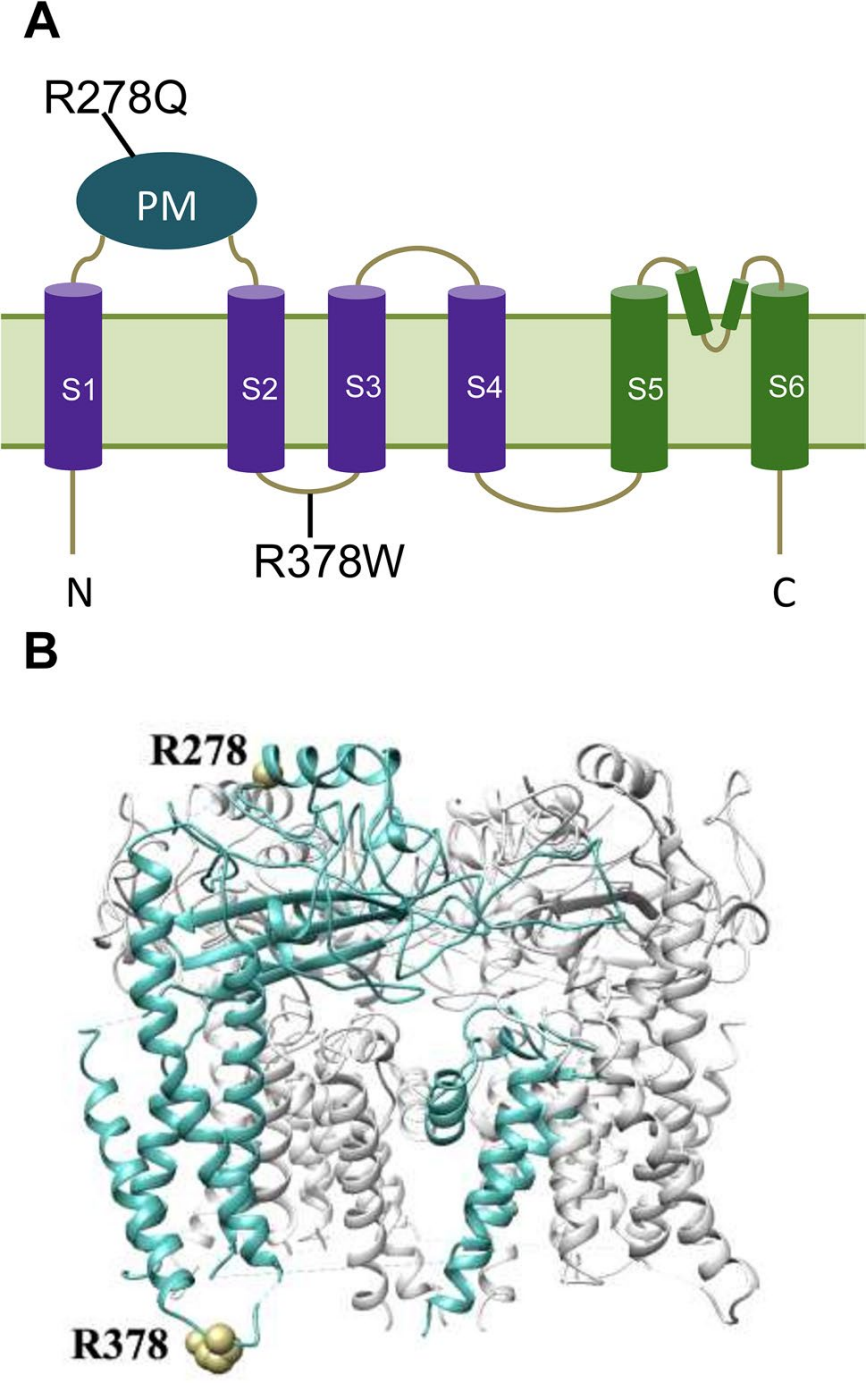
Schematic representation of TRPP3 topology in the cellular membrane and location of R278Q and R378W variants. (**A**) Cartoon illustrating the membrane topology of a TRPP3 monomer. S1–S4 transmembrane segments constitute the voltage sensor domain (VSD) and S5–S6 contribute to the pore region. The polycystin mucolipin domain (PMD) locates in the extracellular loop between S1 and S2 transmembrane segments. The variants are located in the PMD (R278Q) and in the intracellular linker between the S2 and S3 α-helices (R378W). (**B**) Precise location of residues R278 and R378 according to the human TRPP3 cryo-EM structure of the tetrameric channel (Worldwide Protein Data Bank (6DU8) and Electron Microscopy Data Bank (8912))^19^. Affected residues are only shown in the TRPP3 monomer coloured in cyan.

The allele frequency increase of functional genetic variants is the major driver of human population adaptation to the wide variety of environments by adaptive (positive) selection. In a recent study of positive selection in several populations from Ethiopia from our lab (Walsh et al., in press) several interesting signals were uncovered using, by one hand, the SFselect software^37^ based in detecting changes in the site frequency spectrum, which uncovers mainly ancient signals and by the other hand, the iHS test^38^, based in the extension on linkage disequilibrium, which detects recent events that are mainly population-specific. One of the top signals of positive selection of the iHS analysis (showing also a high population differentiation as measured with the F_st_ index) was a region in the long arm of chromosome 10 that contains the *TRPP3* gene (Fig. 1A). The signal is not found in other populations such as the European (CEU) or the West African (YRI) populations from 1000 Genomes Project. We also checked the PopHuman selection browser^39^ for signals of selection in a set of 26 worldwide populations from the Phase 3 1000 Genomes Project and did not find evidence of positive selection in any of them. Thus, this signal is found exclusively in a Nilo-Saharan-speaking population from Ethiopia, the Gumuz. This population is not affected by the extensive admixture from the Levant that is found in other Ethiopian populations such as the Amhara, Oromo, Somali and Wolayta^40^. In the *TRPP3* gene, there are two non-synonymous variants with high iHS values, and a very strong population differentiation. In the Gumuz population these exonic variants, rs17112895 and rs7909153, are present in its derived form (T and A, respectively) both at a frequency of 0.71, much higher than in other African populations, with allele frequencies decreasing towards the west (Fig. 1B and Table 1A–C); in all non-African populations, the allele frequency of the derived allele is zero. These two variants encode for non-synonymous genetic variants (R278Q and R378W) located in the most external α-helix of the extracellular polycystin mucolipin domain (PMD) and in the intracellular linker between transmembrane segments S2 and S3 at the voltage sensor domain (VSD) of the TRPP3 channel, respectively (Fig. 2). There is a strong linkage disequilibrium in the region, even considering that these are sub-Saharan populations, characterized with general low levels of linkage disequilibrium^41–43^. In fact, pairwise linkage disequilibrium measurements between both variants indicate that they are in strong linkage disequilibrium (r^2^ = 1 and D′ = 1). It is interesting to note that the two variants have an ancient age within the spectrum of the age of human SNPs. According to the Genealogical Estimation of Variant Age^44^, rs7909153 and rs17112895 would have an age of 290,000 and 683,000 years respectively (with a generation time of 25 years). The interesting points here are that they have different ages and that both are much older than the Out of Africa expansion of modern humans. Together with the fact that both variants are found at lower frequencies in other African populations suggests that selection acted in standing variation that was previously neutrally segregating in Africa.

**Table 1:** Summary of substitutions rs17112895 and rs7909153.

rsID	Genomic position (hg19)	Ancestral nucleotide	Derived nucleotide	Amino acid site	Ancestral amino acid	Derived amino acid	Gumuz	Amhara	LWK	YRI	MSL	CEU
**(A)**												
rs17112895	102057262	C	T	278	R	Q	0.71	0.25	0.22	0.18	0.13	0
rs7909153	102056790	G	A	378	R	W	0.71	0.27	0.25	0.21	0.15	0

rsID	Genomic position (hg19)	Ancestral nucleotide	Derived nucleotide	Gumuz fraction of ancestral homozygotes	Gumuz fraction of heterozygotes	Gumuz fraction of derived homozygotes						
**(B)**												
rs17112895	102057262	C	T	0.04	0.5	0.46						
rs7909153	102056790	G	A	0.04	0.5	0.46						

Haplotype	Amino Acid	Gumuz	Amhara	YRI	LWK	MSL	CEU					
(**C**)												
A,T	Q278, W378	0.71	0.21	0.16	0.04	0.13	0					
G,T	R278, W378	0	0	0.01	0.13	0.01	0					
A,C	Q278, R378	0	0.02	0.03	0.19	0.03	0					
G,C	R278, R378	0.29	0.77	0.8	0.64	0.84	1					

(**A**) Genomic location and allele frequencies in several populations (Gumuz, Amhara, Somali, Wolayta from Ethiopia, YRI: Yoruba from Nigeria, LWK: Luhya from Kenya, MSL: Mende from Sierra Leone, CEU: Europeans). (**B**) Fraction of Gumuz individuals homozygous and heterozygous for both substitutions. (**C**) Haplotype frequencies of both substitutions in worldwide context.

### Electrophysiological analysis reveals impaired function of double mutant TRPP3

**Figure 3:**
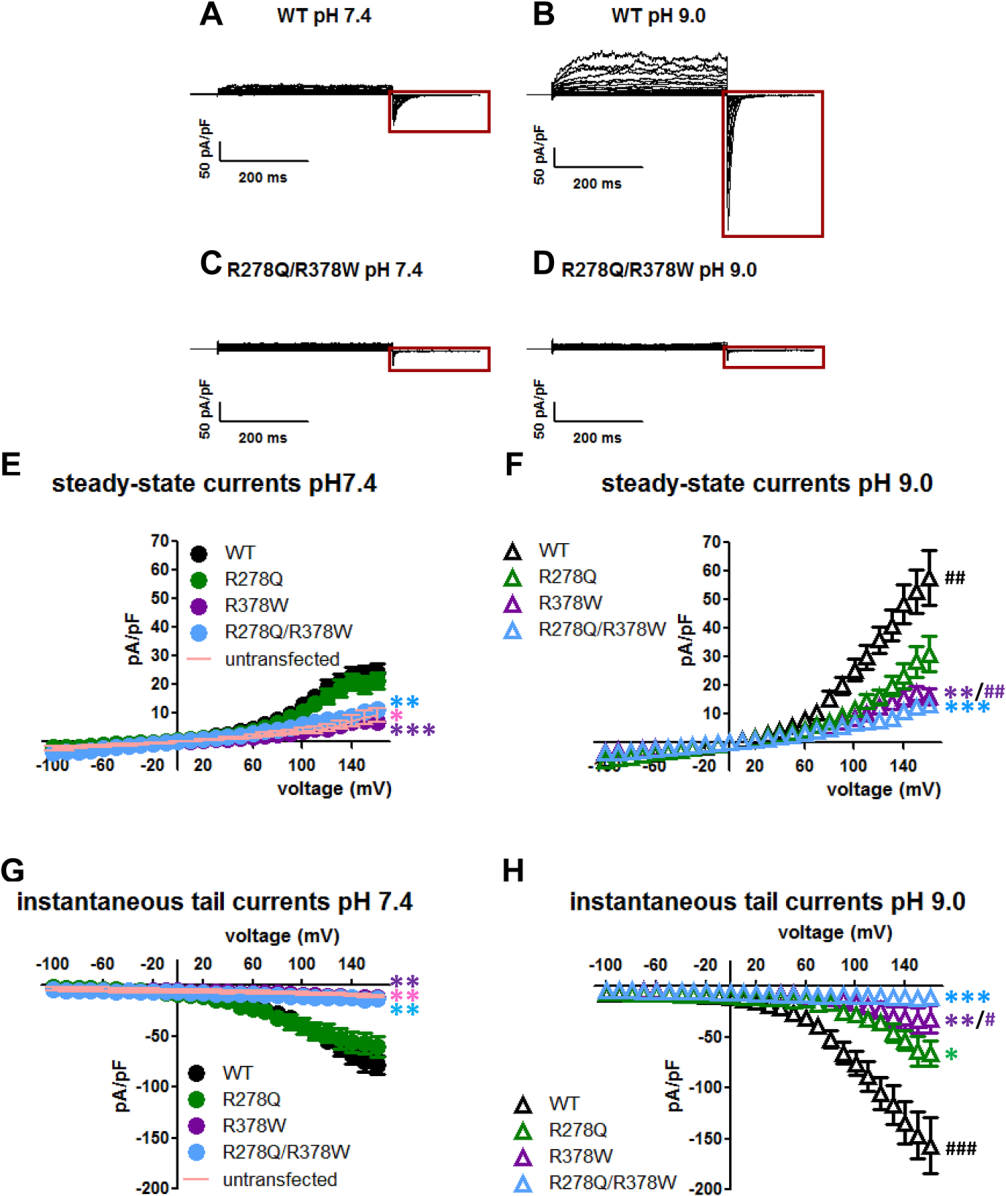
Loss of TRPP3 function induced by mutations R278Q and R378W. (**A–D**) Representative whole-cell currents from HEK293 cells co-transfected with cDNAs encoding PKD1L3 and wild-type (WT) or double mutant (R278Q/R378W) TRPP3-GFP and exposed to either extracellular pH 7.4 or pH 9.0, as indicated. Currents were elicited by step pulses from − 100 to + 160 mV in 10 mV increments with a postpulse to − 100 mV, and normalized by cell size (membrane capacitance). Red boxes show tail currents after membrane repolarization to − 100 mV. (**E–H**) Average current–voltage (I–V) relationships for steady-state currents (**E**,**F**) and instantaneous tail currents (**G**,**H**) at each test pulse in untransfected HEK293 cells and HEK293 cells expressing PKD1L3 and wild-type (WT) or mutants (R278Q, R378W, R278Q/R378W) TRPP3-GFP and exposed to either extracellular pH 7.4 or pH 9.0, as indicated. Area under the I–V curves (AUCs) were calculated for statistical analysis (only values at positive voltages were taken under consideration for steady-state I–V curves). Obtained AUC values for steady-state currents were: control untransfected cells at pH 7.4 (pink line, n = 6) 533.15 ± 145.3; WT at pH 7.4 (black circle, n = 19) 1662.6 ± 233.9; WT at pH 9 (black triangle, n = 17) 3213.9 ± 444.2; R278Q at pH 7.4 (green circle, n = 9) 1375.6 ± 184.3; R278Q at pH 9 (green triangle, n = 10) 1663.9 ± 291.7; R378W at pH 7.4 (purple circle, n = 5) 492 ± 72.5; R378W at pH 9 (purple triangle, n = 6) 1102.3 ± 176; R278Q/R378W at pH 7.4 (blue circle, n = 10) 620.3 ± 100.4; R278Q/R378W at pH 9 (blue triangle, n = 6) 856.1 ± 132.5. Obtained AUC values for instantaneous tail currents were: control untransfected cells at pH 7.4 (pink line, n = 6) − 1689 ± 359.4;WT at pH 7.4 (black circle, n = 19) − 6034.2 ± 649.3; WT at pH 9 (black triangle, n = 17) -10,882.7 ± 1437.6; R278Q at pH 7.4 (green circle, n = 9) − 5563.5 ± 1010.1; R278Q at pH 9 (green triangle, n = 10) − 5034 ± 920.5; R378W at pH 7.4 (purple circle, n = 5) − 1860 ± 218.1; R378W at pH 9 (purple triangle, n = 6) − 2992 ± 538.2; R278Q/R378W at pH 7.4 (blue circle, n = 10) − 2005.8 ± 229.2; R278Q/R378W at pH 9 (blue triangle, n = 6) − 1979.2 ± 117.5. ^#^P < 0.05, ^##^P < 0.01 and ^###^P < 0.001 (when compared to the corresponding TRPP3 channel at pH 7.4; one-tail Student’s t-test or one-tail Mann–Whitney *U*-test, as appropriate); *P < 0.05, **P < 0.01 and ***P < 0.001 (when compared to the WT channel at the corresponding extracellular pH condition; Kruskal–Wallis, followed by Dunn post hoc test).

Next, we aimed to characterize the functional consequences of the two TRPP3 non-synonymous genetic variants (R278Q and R378W) by comparing channel activity of the wild-type (WT) TRPP3 channel with that of channels carrying either the double substitution (R278Q/R378W) or the single substitutions (R278Q or R378W). For that purpose, we performed whole-cell patch-clamp recordings on GFP positive HEK293 cells co-transfected with PKD1L3 and GFP-fused TRPP3 channels (TRPP3-GFP). We measured robust channel activity in response to voltage changes in cells expressing WT TRPP3. As previously reported^20^, they exhibited outwardly rectifying currents with large tail currents after repolarization to − 100 mV at extracellular pH 7.4, whereas no such currents were recorded from untransfected HEK293 cells (Fig. 3A,E,G and Supplementary Fig. S1A,B online). Besides, recordings at extracellular pH 9.0 revealed largest TRPP3 steady-state and tail currents (Fig. 3B), as reported before for HEK293 cells expressing only TRPP3 channels in the absence of PKD1L3^22^. The average current–voltage relationships for WT steady-state currents and tail currents at each test pulse and pH condition are shown in Fig. 3E–H. Furthermore, we also found that at extracellular pH 9, the deactivation time course of WT TRPP3 tail currents was accelerated when compared to the extracellular pH 7.4 condition (see Supplementary Fig. S2A,C online), another known effect of alkalinisation on TRPP3 tail currents^22^.

The presence of the double substitution R278Q/R378W reduced the amplitude of both TRPP3 steady-state and tail currents recorded at pH 7.4 when compared to the WT channel (Fig. 3C,E,G). Moreover, unlike the response of the WT channel to alkalinisation, currents through the double variant TRPP3 channel were not enhanced at the extracellular pH 9 condition (Fig. 3D,F,H).

When recorded at pH 7.4, TRPP3 steady-state and tail currents through the R278Q mutant channel were of similar magnitude to those found for the WT channel (Fig. 3E,G and Supplementary Fig. S1D online). However, no significant increase in R278Q TRPP3 current amplitude neither faster deactivation kinetic was observed at pH 9 (Fig. 3F,H and Supplementary Fig. S1E, S2B,D online).

The presence of the single substitution R378W also impaired TRPP3 channel activity at pH 7.4, as observed for the double substitution R278Q/R378W (Fig. 3E,G and Supplementary Fig. S1F online). The magnitude of the whole-cell currents recorded from cells expressing these mutant channels was indistinguishable from that obtained in untransfected cells (Fig. 3C,E,G and Supplementary Fig. S1A,F online). Yet, R378W TRPP3 steady-state and tail currents were slightly but significantly largest at pH 9 (Fig. 3F,H, and Supplementary Fig. S1G online).

**Figure 4:**
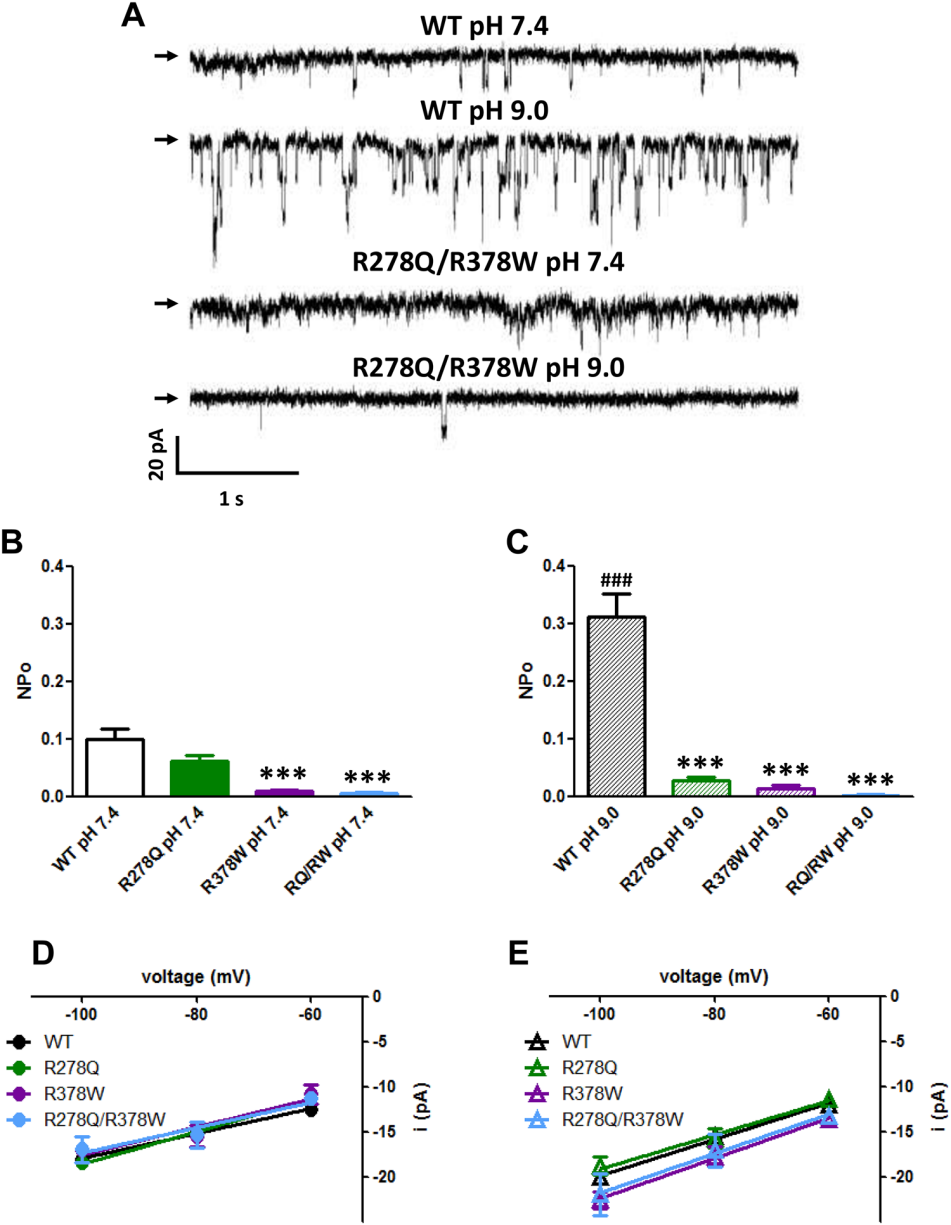
Effect of mutations R278Q and R378W on spontaneous TRPP3 single-channel activity. (**A**) Representative single-channel activity of wild-type (WT) or double mutant (R278Q/R378W) TRPP3 channels in whole-cell recordings obtained at negative membrane potential (− 80 mV) from HEK293 cells co-transfected with cDNAs encoding PKD1L3 and wild-type (WT) or double mutant R278Q/R378W TRPP3-GFP, and exposed to either extracellular pH 7.4 or pH 9.0, as indicated. Arrows indicate the zero current level. (**B**,**C**). Average channel activity (NP_O_) in whole-cell recordings obtained at negative membrane potentials (− 60, − 80 and − 100 mV) from HEK293 cells co-transfected with cDNAs encoding PKD1L3 and wild-type (WT) or mutants (R278Q, R378W, R278Q/R378W) TRPP3-GFP, and exposed to either extracellular pH 7.4 or pH 9.0, as indicated. ^###^P < 0.0001 (when compared to WT channels at pH 7.4; one-tail Mann–Whitney *U*-test); ***P < 0.001 (when compared to the WT channel at the corresponding extracellular pH condition; Kruskal–Wallis, followed by Dunn post hoc test). (**D**,**E**) Average single-channel current amplitudes and slope conductances at negative potentials (between – 60 and − 100 mV) obtained in whole-cell recordings from HEK293 cells co-transfected with cDNAs encoding PKD1L3 and wild-type (WT) or mutants (R278Q, R378W, R278Q/R378W) TRPP3-GFP, and exposed to either extracellular pH 7.4 or pH 9.0, as indicated. No significant differences were found between WT and mutant TRPP3 channels or between pH conditions (one-way ANOVA, P = 0.1974). Single-channel conductance values (in pS) for the different TRPP3 channels were: WT at pH 7.4 (black circle, n = 16) 137.8 ± 16.3; WT at pH 9 (black triangle, n = 14) 201.7 ± 20.1; R278Q at pH 7.4 (green circle, n = 9) 180 ± 13.7; R278Q at pH 9 (green triangle, n = 7) 189.4 ± 35.3; R378W at pH 7.4 (purple circle, n = 3) 155.9 ± 35.6; R378W at pH 9 (purple triangle, n = 7) 222.7 ± 30.4; R278Q/R378W at pH 7.4 (blue circle, n = 4) 139.5 ± 46.3; R278Q/R378W at pH 9 (blue triangle, n = 4) 217.9 ± 73.4.

Because of the low open probability of TRPP3 at hyperpolarized potentials and the large amplitude of the single channel currents it is possible to record spontaneous single-channel activity in the whole-cell configuration^20,22^, which was never observed in untransfected HEK293 cells (see Fig. 4 and Supplementary Fig. S3 online). Under these experimental conditions, we observed that the alkaline condition (pH 9) only increased the open probability (NP_O_) of the WT channel (Fig. 4A–C). Besides, as observed for macroscopic TRPP3 steady-state and tail currents, WT and the R278Q channels showed similar activity only at extracellular pH 7.4, whereas the open probability of R378W and R278Q/R378W channels is lower at both neutral (pH 7.4) and alkaline (pH 9) conditions when compared to the WT channel (Fig. 4A–C and Supplementary Fig. S3 online). As reported before^22^, WT TRPP3 showed similar single-channel conductance at extracellular pH 7.4 and 9, and it was not altered in the TRPP3 channels with the substitutions (Fig. 4D,E).

**Figure 5:**
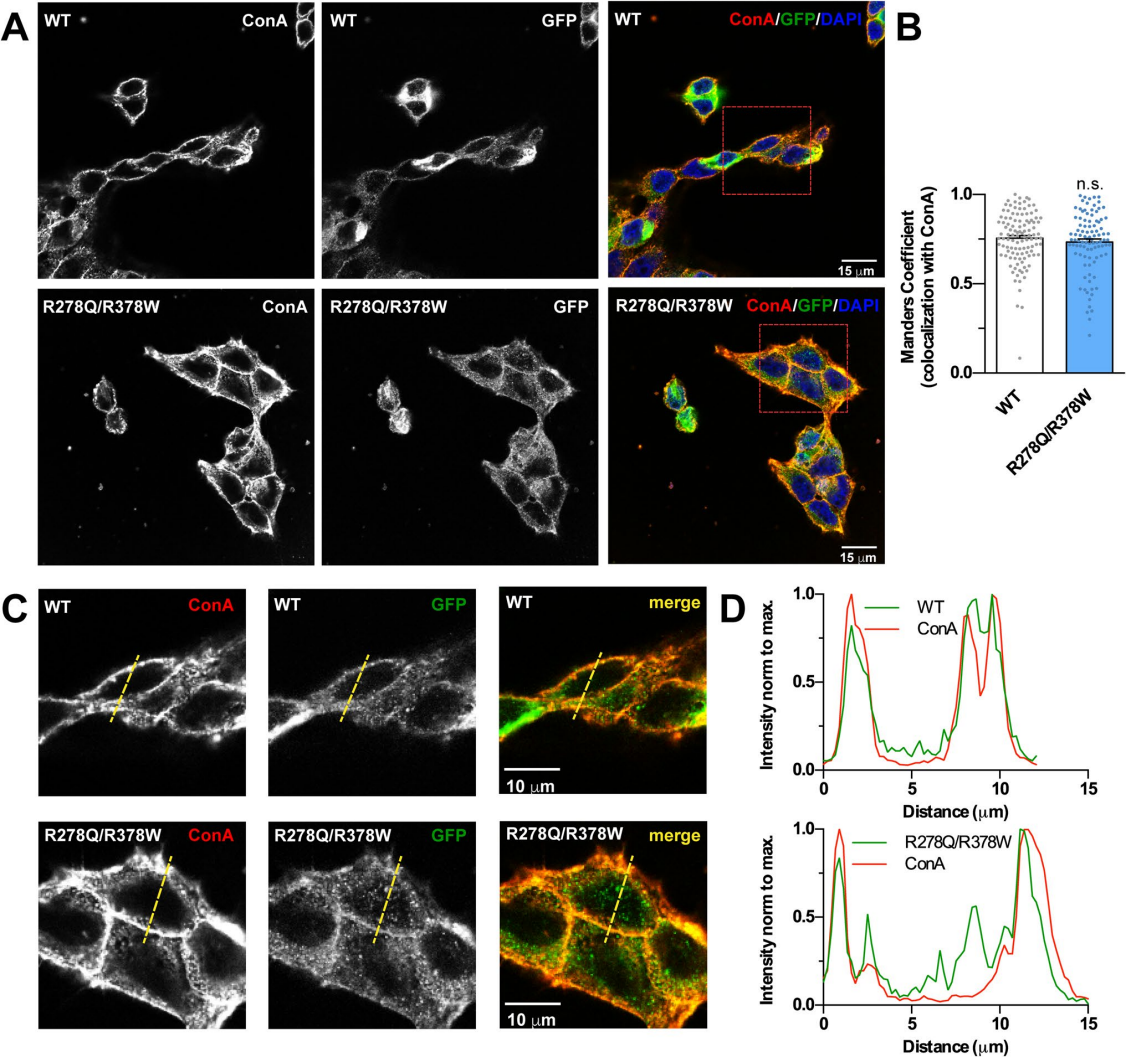
Substitutions R278Q and R378W do not modify the plasma membrane expression level of heterologously expressed TRPP3 channels. (**A**) Confocal images (single z plane) of HEK293 cells co-transfected with cDNAs encoding PKD1L3 and wild-type (WT, top) or double mutant (R278Q/R378W, bottom) TRPP3-GFP channels (middle panels). Cell membranes were stained with ConA-Rhodamine (left) and the overlaid image is shown on the right. Red-dashed boxes indicate the zoomed areas in (**C**). Scale bar 15 µm. (**B**) Manders colocalization coefficient indicating the fraction of TRPP3-channel WT and R278Q/R378W colocalizing with plasma membrane ConA-Rhodamine. Double mutant (n = 97) does not show significant differences when compared to the wild-type (n = 112) TRPP3 channel (Mann–Whitney test, P = 0.5158). (**C**) Zoomed images of the area indicated in A showing two cells with similar expression levels of wild-type (WT, top images) and double mutant (R278Q/R378W, bottom images) TRPP3-GFP channels. (**D**) Normalized pixel intensity profiles of crossed sections indicated by the yellow dashed line in C for WT (top) and double mutant (bottom) TRPP3 channels.

To verify that the decrease in channel activity produced by the double substitution R278Q/R378W was due to a functional alteration of the channel and not to lower presence of the protein in the cell membrane, we evaluated the level of co-localization of both, the ectopically-expressed WT and mutated TRPP3-GFP channels, with the plasma membrane marker concanavalin A. Confocal imaging analysis indicates that both the WT and the TRPP3 proteins with the substitutions are expressed similarly in the plasma membrane (Fig. 5).

Altogether, in agreement with the extreme Combined Annotation Dependent Depletion (CADD) scores, rs17112895 for R278Q and rs7909153 for R378W, have CADD scores of 23 and 24.8 respectively, values that suggest potentially very strong functional effects at protein level. In fact, our data indicate that both genetic variants alone and in combination, exert loss-of-function effects on TRPP3 channels.

After coexpression of WT and double mutant R278Q/R378W channels we did not observe a dominant negative effect of the double mutation (see Supplementary Fig. S1B,C,H,I and S3 online). However, given the high proportion of homozygotes in the Gumuz population carrying the two mutations (Table 1B) our functional characterization of the mutant channel remains relevant.

### Further evidence of TRPP3 mutant impaired function through protein 3D modelling

We next explored by 3D structural modelling the structural modifications driven by the R378W substitution, that changes the charged side-chain of arginine by the aromatic chain of tryptophan. This stabilizes the helix formed in the linker between S2 and S3 in the closed-channel conformation by locating the side-chain upwards towards the inner membrane (see model in Supplementary Fig. S4 online). Besides, the comparison of the structures of the wild type and mutant form in open conformation (see Supplementary Fig. S5 online) shows that the connecting helix between S2 and S3 is unfolded, with a movement of helix S4 that orients K455 towards D390 in the wild type, according to^45^. The structural analysis of the models shows a similar distribution of contacts at hydrogen bonding distance (H-bond, considering the maximum acceptable distance to form the hydrogen bond at 3.5 Å), between D390 and K452 and between D390 and K455, for both mutant and wild type forms (see Supplementary Fig. S6 online). Also, the distribution of distances between D390 and K455 when they are not at H-bond distance has an average around 8.7 Å, but the deviation is larger for R378W (2.3 Å) than for the wild type form (1.9 Å), which produces a strong significant difference (Mann–Whitney *U*-test P-value of 1.1e^−106^). Furthermore, wild type and R378W forms have a significant different distribution of the contacts between D390 and K452 when they are not at H-bond distance (with a Mann–Whitney *U*-test P-value of 7.3 e^−54^). The capacity to form a hydrogen bond between D390 and K452 is more often lost in the wild type form than in the R378W form. This is reflected by the ratio of H-bonds D390-K452 preserved (49% for the wild type versus 57% for the mutant form R378W). These trends confirm that while in the wild type form the hydrogen bond swaps from D390-K452 to D390-K455 when opening the channel, in the R378W mutant the hydrogen bond is more restrained and preserves the closed conformation, which implies the loss-of-function.

### Phenotypic analysis in human taste sense

After demonstrating that both substitutions impair the TRPP3 activity, we next examined the putative phenotypic differences for the main function initially associated to the gene, sour tasting, between individuals carrying the derived or the ancestral variants. For that, we recruited a total of 44 individuals of Western African (13), Ethiopian (13) and European (18) ancestry, and genotyped them for both *TRPP3* variants (not all individuals could be genotyped, see Table 2A,B and Supplementary Table S1 online) from a saliva sample. The Ethiopian sampled populations were of Amhara and Oromo ancestry, none of the individuals had a Gumuz ancestry (a population we cannot access for the test). For the phenotypical analysis, we determined their sour taste recognition threshold following the described method^46^. Briefly, the individuals were asked to blindly taste solutions of citric acid at different concentrations and fill a questionnaire related to qualitative sour tasting foods. Due to differential TRPP3 channel activity through alkalinisation^22^, we also determined the saliva pH of some of the individuals.

**Table 2:** Summary of the sampled individuals for the sour taste phenotypic analysis.

Population	Chromosomes	Males/females	Genotyped chromosomes	Q278 frequency	W378 frequency	278^R/R^ 378^R/R^	278^R/Q^ 378^R/W^	278^Q/Q^ 378^W/W^	Mean RT (mM)	Mean pH
**(A)**										
WA	26	7/6	26	0.31	0.31	9	4	0	0.240	–
ET	26	9/4	24	0.25	0.25	10	1	1	0.225	6.9
EU	36	6/12	30	0	0	15	0	0	0.250	6.6

	Samples	Males/females	Mean RT (mM)	Mean pH						
**(B)**										
278^R/R^ 378^R/R^	34	15/19	0.24	6.8						
278^R/Q^ 378^R/W^	5	4/1	0.24	6.25						
278^Q/Q^ 378^W/W^	1	1/0	0.20	7						

(**A**) Number of chromosomes per ancestry, Ethiopian (ET), West African (WA) or European (EU), number of males and females, frequency of the substitutions, number of individuals carrying the three genotypes and mean recognition threshold (RT, citric acid in mM) and mean saliva pH. (**B**) Number of samples, number of males and females and mean recognition threshold and mean saliva pH of individuals carrying the three possible genotypes.

Among the genotyped individuals, we found 34 homozygous for the ancestral alleles (278^R/R^ 378^R/R^), 1 double homozygous for the derived alleles (278^Q/Q^ 378^W/W^) and 5 double heterozygous (278^R/Q^ 378^R/W^) indicating that both loci are in strong linkage disequilibrium (Table 2B). All individuals (6) carrying a derived state of an allele are of African origin, as expected by the general population allele frequencies that have a zero allele frequency outside Africa. The limited number of individuals carrying the derived alleles mirrors the low population allele frequencies of the variants within African (non-Gumuz Ethiopians and West Africans) populations.

**Figure 6:**
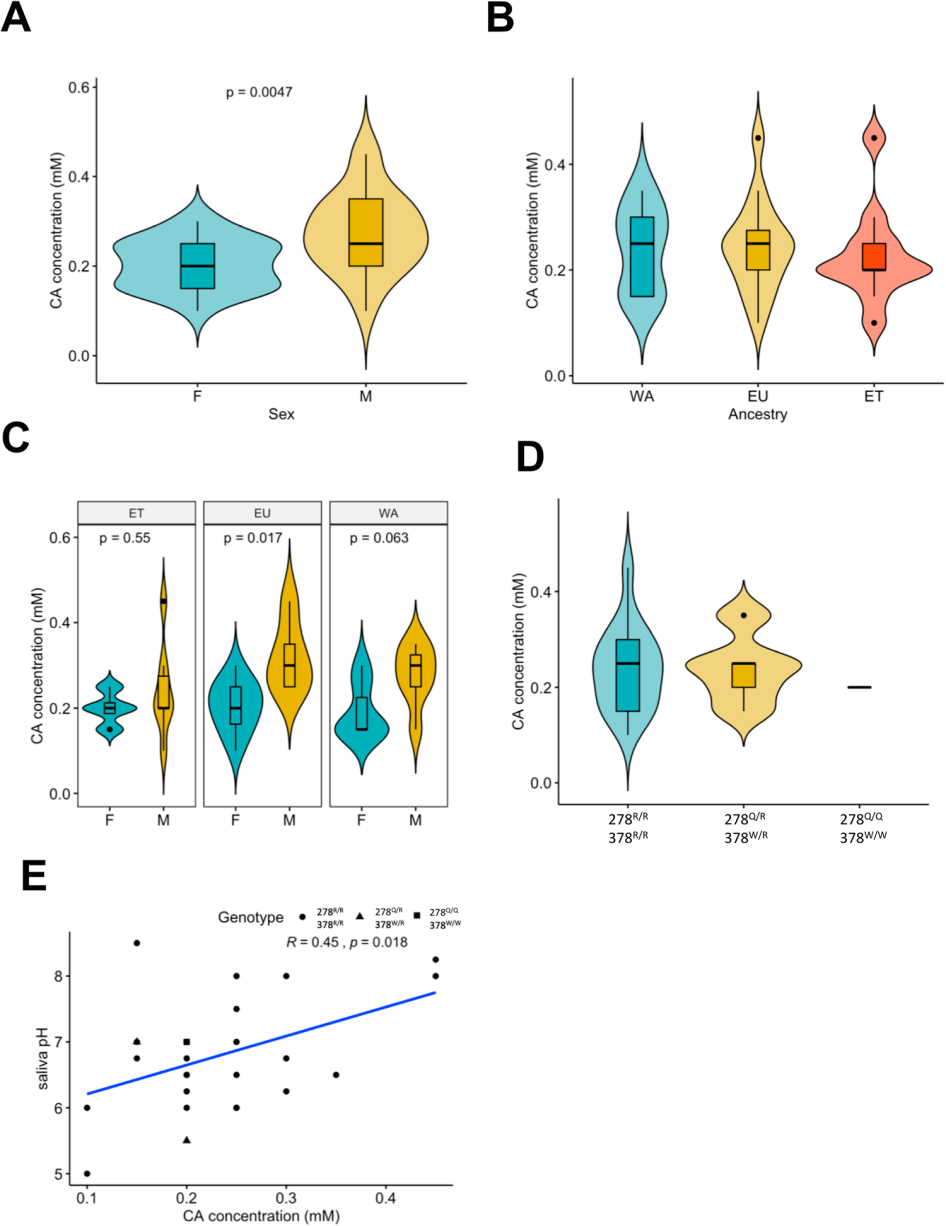
Genotype–phenotype analysis of the sour recognition thresholds. (**A**) Significant differences between males and females of sour recognition threshold (Wilcoxon test W = 116.5 p-value = 0,005). (**B**) Sour recognition threshold of the three sampled populations, Ethiopia (ET), West Africa (WA), Europe (EU). No significant recognition threshold differences are observed between populations after applying an analysis of covariance of ranked data controlling for sex and saliva pH. (**C**) Sour recognition thresholds of the three studied populations stratified by sex. Wilcoxon test was performed to test for differences between sexes within a same population and p-values were adjusted with holm correction. (**D**) Genotype analysis of individuals and sour recognition thresholds. We found individuals with both substitutions at an ancestral homozygous state (278^R/R^ 378^R/R^), heterozygous state (278^Q/R^ 378^W/R^) or derived homozygous state (278^Q/Q^ 378^W/W^). No significant differences are found between the different genotypes after applying an analysis of covariance of ranked data controlling for sex and saliva pH. (**E**) Positive correlation between saliva pH and sour recognition threshold.

The common saliva pH ranges from 6.2 to 7.6^47^. For the analysed cohort, we correlated the pH and the recognition threshold for sour taste. We observed a significant positive correlation between saliva pH and the sour recognition threshold (Fig. 6E, Pearson’s r = 0.45, p-value = 0.018). We also report significant differences between males and females of sour recognition thresholds (Fig. 6A,C). Nonetheless, no significant sour recognition differences were observed between the three populations after an analysis of covariance controlling for sex and saliva pH (Fig. 6B, F = 1.43, df = 2, p-value = 0.26) or between the three different observed genotypes (Fig. 6D, F = 1.36, df = 2, p-value = 0.28). The limited sample size of individuals carrying the derived alleles does not allow a clear result, but at this level we cannot claim any threshold difference related to the presence or not of the two TRPP3 substitutions.

## Discussion

We report the functional consequences on TRPP3 channel activity of two non-synonymous genetic variants (R278Q and R378W) that we identified as positively selected amino acid substitutions in the Gumuz population from Ethiopia. Our results show that both genetic variants lead to TRPP3 loss-of-function without alteration of channel trafficking to the plasma membrane, which is similar for both wild-type (WT) and double mutant R278Q/R378W TRPP3 channels. Substitution R278Q, at the most external α-helix of the extracellular polycystin mucolipin domain (PMD), impairs TRPP3 activation by alkaline pH. And substitution R378W, at the intracellular linker between transmembrane domains S2 and S3 of the voltage sensor domain (VSD), strongly reduces channel activity at both neutral (pH 7.4) and alkaline (pH 9) conditions but still show some degree of activation in response to alkalinisation. Double substitution R278Q/R378W TRPP3 channels show both nearly no activity at neutral pH and impaired modulation by alkaline pH.

Concerning TRPP3 structure–function relationship, our results reveal an important role of the PMD in channel sensitivity to pH. Because of its structural organization, PMD seems to facilitate the assembly of polycystin domains in the tetrameric channel and maintain their stability^18,19^. In this process it seems to be involved the PMD residue E290 (located at the same external α-helix containing the R278Q substitution), which forms a hydrogen bond with residue N176 of the adjacent PMD in the tetramer (see Supplementary Fig. S7 online). Besides, PMD interacts with the pore domain and it is in close structural relationship with the VSD (in particular the S3 transmembrane segment and the extracellular S3-S4 helical region)^18,19^. With regards of TRPP3 modulation by pH, from the comparison of structural data obtained for TRPP3 and another pH sensitive channel (TRPML3) at neutral and acid pH conditions, it has been proposed that the TRPP3 acid-evoked off-response may be due to the release of an inhibition produced by the binding of H^+^ to certain negatively charged residues of the PMD (E135, D143 and D156) after acid washout^18,48^. Thus, TRPP3 activation in response to alkalinisation would be the consequence of conformational changes in the PMD region interacting with H^+^ that in turn transduce a signal affecting both accessibility to the external pore region and VSD movement. Accordingly, we can speculate that the neutralization of the positive charge produced by the R278Q substitution in the upper α-helix of the PMD might modify its global structure organization and affect PMD assembly and steadiness, and/or the relative position of the negatively charged amino acids interacting with H^+^ (for instance, residue D156 located in the α-helix immediately below the one containing the R278Q substitution)^18,19^. In this respect, it is worth to notice that in this second α-helix, residue D167 is close to residue R278^19^ and the RQ change might alter a possible electrostatic interaction among them, modify the relative position of these two α-helices and therefore affect the location of residue D156 involved in H^+^ binding (see Supplementary Fig. S7 online). Such conformational alteration might explain the impaired activation in response to alkaline pH 9 of TRPP3 channels carrying the R278Q variant.

The polycystin domains of TRPP3 share similar structural features and high degree of homology to PKD2 (also termed TRPP2)^18,19,49^, that together with PKD1 play crucial roles in autosomal dominant polycystic kidney disease (ADPKD), a common and potentially lethal disorder resulting in major renal disorders. Loss-of-function mutations of either PKD1 or PKD2 account for the majority of ADPKD cases^50^. Multiple ADPKD missense mutations affect the polycystin domain of PKD2 [The Autosomal Dominant Polycystic Kidney Disease Database (PKDB)]. None of them are located at the equivalent TRPP3 external α-helix containing the R278 residue (which, along with D167, is conserved in PKD2, corresponding to R399 and D289). However, there are three substitutions of conserved amino acids nearby, according to the 3D channel structure: W280R, Y292C and W414G (W158, Y170 and W293 in TRPP3, respectively)^51–53^. The first two changes are located at the α-helix close to the most external one containing the R399/R278 residue (in PKD2 and TRPP3, respectively). The third change affects the first amino acid of the loop that follows at this external α-helix. The three PKD2 mutations may have an important effect on the correct folding of this region of the polycystin domain, resulting in a hampered protein function that leads to polycystic kidney disease. These findings altogether suggest a relevant role of this polycystin domain area around the most external α-helix in the correct function of both PKD2 and TRPP3 channels.

TRPP3 channel gating in response to voltage or other physiological stimuli seems to involve transitions from closed to open triggered by pore coupling with VSD motion. Such coupling would be mediated by interactions between residues at the S5 and S4 α-helices of adjacent monomers, leading to the widening of both the lower and the upper gates^18^. Two lysines at the cytoplasmic end of S4 (K452 and K455), which are potentially stabilized by amino acids from S1 (Y107), S2 (Y366), and S3 (D390), appear to explain TRPP3 voltage-dependent properties^18^. In agreement with this view, charge-neutralizing mutations K452Q and K455Q strongly weaken TRPP3 voltage dependence, and these mutant channels do not reach maximal activation even upon very strong depolarisation levels (up to + 300 mV)^54^. By structural modelling it has been suggested that the functional impairment after neutralization of these two positively charged amino acids is due to a marked restriction of the voltage sensor movement^54^. Accordingly, more recent structural modelling shows that these two gating charges (K452 and K455) can generate S4 transitional and lateral movements in response to membrane depolarization that in turn will be coupled to pore opening. During this VSD motion, the negatively charged D390 of S3 segment would stabilize K452 in the closed state and K455 in the open state. Furthermore, Y366 would interact sequentially first with K452 (in the deactivated state) and then with K455 (in the activated state) to provide the required energy for channel opening^45^. This coupling between VSD movement and pore opening seems to be a general mechanism for TRPP3 activation applying also for other physiological stimuli. Thus, neutralization of one of these two gating charges strongly impairs channel gating regardless of the stimulus employed (voltage, hypoosmotic shock or temperature)^45^.

It is remarkable that all VSD residues mentioned above are also conserved in PKD2 (K572 and K575 at S4, Y227 at S1, Y487 at S2 and D511 at S3)^18,49^. Moreover, loss-of-function mutation D511V is a frequent pathogenic missense variant linked to ADPKD^55,56^. Structural analysis of both PKD2 and TRPP3 channels suggest that the neutralization of this negatively charged residue at S3 by valine substitution most probably alter the stabilization of S4 lysine residues and perturb both VSD structure and function, and thus subsequent pore gating in response to voltage changes^18,49^. The R378W substitution we found at high frequency in the Gumuz population, by affecting the S2–S3 intracellular linker may well have a similar effect by altering VSD conformation, in particular the correct position of the S3 segment, and thus impair the stabilization of S4 lysines. The structural analysis of R378W variant shows the preference of the closed conformation over the open. Although the models are not able to capture the full motion of S4 described by^45^, they can be used to unveil notable differences between the wild type form and the R378W mutation, suggesting the larger stability of the D390-K542 hydrogen bond and the helix conformation of the linker S2-S3 in the mutant form, which may hamper the motion towards the open state. This might be the underlying cause of the strong reduction in channel activity we observed for TRPP3 channels carrying the R378W variant.

Given the strong linkage disequilibrium of the two ancestral and derived alleles, if PKD1L3/TRPP3 channel complex were somehow involved in sour taste detection^25–28^ it would be expected that individuals carrying the loss-of-function R278Q/R378W genetic variants had impaired the recognition of acidic stimuli across the tongue. However, we did not find significant sour recognition differences between genotypes (R278/R378 double homozygous, Q278/W378 double homozygous, or double heterozygous). This conclusion is limited by the low number of R278Q/R378W carriers (one in homozygosis and five in heterozygosis) in the group of Africans participating in the sour taste sensitivity test (as none of them had a Gumuz ancestry). Yet, the fact that all six R278Q/R378W carriers could detect acidic stimulation without apparent loss, argues against both any involvement of PKD1L3/TRPP3 in sour detection and the role of such physiological process in the evolutionary positive selection we report here. Altogether, these results agree with the current idea that considers the proton channel OTOP1 as the real sour sensor in TRPP3-expressing, acid-detecting taste receptor cells^32–36^.

Several functional roles have been proposed for TRPP3. Thus, this channel interacts and co-localizes with β_2_-adrenergic receptor (β_2_AR) on the primary cilia of neuronal cells in mice brain, where TRPP3 deficiency leads to the loss of β_2_AR on neuronal primary cilia, reduction of brain cAMP levels, increased neuronal excitability and higher susceptibility to seizure induced by the chemoconvulsant pentylenetetrazol^57^. Besides, in zebrafish, Trpp3 is expressed in cerebrospinal fluid-contacting neurons and required for the responses to pressure and maintenance of spine curvature. Indeed, zebrafish that do not express Trpp3 present an exaggerated spine curvature similar to human kyphosis^21^. Lastly, it has been shown that although TRPP3 knockout mice had a decreased cytosolic and mitochondrial calcium entry in cardiomyocytes when they were fed with a normal-salt diet, TRPP3 absence results in a mitochondrial calcium overload in muscle cardiac cells of animals under high-salt diet. Such mitochondrial dysregulation in murine cardiomyocytes exacerbates high-salt diet-induced cardiac hypertrophy and dysfunction, revealing the protective effect of TRPP3 on cardiac hypertrophy in mice exposed to high-salt loading^58^. Notably, rs17112895 and rs7909153 are marginally associated to heart rate recovery on the GWAS Atlas database^59^ with p-values of 0.00073 and 0.003, respectively, even if their association signals did not pass the genome-wide multiple testing on the original study (describing 23 loci associated with heart rate increase or recovery based on 58,818 UK Biobank individuals)^60^.

Further research on physiological and pathological traits of the Gumuz population at both neurological and cardiovascular levels is therefore needed in order to unveil the reasons behind the strong positive selection of the loss-of-function R278Q/R378W genetic variants at the TRPP3 channel in Gumuz Ethiopians.

## Methods

### Genomic data

The Gumuz individuals analyzed in this study are comprised within the European Genome-phenome Archive (EGA) repository with accession number EGAS00001000238. The dataset consists of five East African populations (Amhara, Oromo, Somali, Wolayta and Gumuz) with 24 individuals each from Pagani et al. 2015. Additional samples from the 1000 Genomes Project (Gibbs et al. 2015) also from Pagani et al. were included to calculate positive selection statistics and allele frequencies. The genome assembly of the data is GRCh37 (hg19).

### Genomic analysis of positive selection

To identify that positive selection has acted on the PKD2L1 gene, this region was investigated after the results presented in Walsh et al. 2020. The linkage disequilibrium-based test iHS was used to detect recent events of selection in the Gumuz population and other populations for comparison (CEU and YRI). The software rehh 2.0 (Gautier et al. 2017) was used to calculate iHS for all the genetic variants with a minor allele frequency higher than 0.05 and excluded a variant from the calculation if a 20 kb gap was found when calculating the extension of the haplotype homozygosity. The mean iHS score (average of iHS scores across SNPs) in each 30 kb window was calculated. To assess the statistical significance of the scores, an iHS critical value was defined after performing extensive genetic neutral simulations. The demographic model implemented in the simulations was a typical three-population model representing Africans, Europeans and Asians (Jouganous et al. 2017). The 99.99th percentile of the neutral distribution of IHS scores was used as critical value, being 2.54.

### cDNA constructs

cDNA of the human TRPP3 wild-type (WT) channel, cloned into the plasmid pCMV6-AC-GFP was obtained from OriGene, Maryland, United States. TRPP3 mutant channels (R278Q, R378W and R278Q/R378W) were generated by site-directed mutagenesis of the human WT TRPP3 cDNA using the QuikChange XL kit (Stratagene), forward (5′-CTTCCAGGATCCCAACAGGGTAGTGCA-3′) and a reverse (5′-TGCACTACCCTGTTGGGATCCTGGAAG-3′) primers for R278Q substitution, and forward (5′-AGCTCCACATTCACTGGCTTCGCTACCT-3′) and a reverse (5′-AGGTAGCGAAGCCAGTGAATGTGGAGCT primers for R378W substitution. cDNA of mouse PKD1L3, cloned into pDisplay vector was a gift from Dr. Yong Yu (Department of Biological Sciences, St. John's University, 8000 Utopia Parkway, Queens, New York, 11439, USA). All cDNA clones were sequenced previously to the study in order to confirm their integrity.

### Heterologous expression

HEK293 cells were transfected using a linear polyethylenimine (PEI) derivative, the polycation ExGen500 (Fermentas Inc., Hanover, MD, USA) as previously reported (eight equivalents PEI/3.3 μg DNA/dish)^61^. Co-transfection was performed using the ratio for GFP-fused TRPP3 (WT or either mutant R278Q, R378W, or R278Q/R378W), and PKD1L3 cDNA constructs of 1:1. For testing possible dominant negative action of the double mutant, cells were co-transfected with WT, R278Q/R378W TRPP3-GFP and PKD1L3 cDNA constructs with a ratio of 1:1:2. Transfected cells were incubated for 24 h and seeded on 35 mm diameter dishes Poly-d-Lysine-coated (Sigma). Experiments were performed 48 h post-transfection at room temperature (22–24 °C).

### Whole-cell patch-clamp recordings and electrophysiological analysis

Patch electrodes had a resistance of 2 MΩ when filled with a pipette solution containing (in mM): 80 CsMES, 20 NaCl, 2 MgCl_2_, 10 HEPES and 5 BAPTA (pH 7.4, adjusted using TrisBase). Osmolarity was adjusted to 300 mOsm/l adding D-mannitol. Recordings were performed at two different extracellular pH values, 7.4 or 9. In the first case, the external bath solution contained (in mM): 150 NaCl, 2 CaCl_2_, 10 HEPES (310 mOsm/l and pH 7.4, adjusted using Tris-base). The basic external bath solution contained (in mM) 150 NaCl, 2 CaCl_2_ and 10 TAPS (310 mOsm/l and pH 9, adjusted using Tris-base). All chemicals were obtained from Sigma-Aldrich.

TRPP3 activity was assessed using the whole-cell configuration of the patch-clamp technique on GFP-positive HEK293 cells 48 h after co-transfection of PKD1L3 with cDNA encoding wild-type (WT), R278Q, R378W, R278Q/R378W or both WT and R278Q/R378W TRPP3 channels. Whole-cell cationic currents were evoked by the application of 400 ms step pulses from − 100 to + 160 mV in 10 mV increments followed by a 200 ms postpulse to − 100 mV. Besides, spontaneous single-channel currents were also recorded in the whole-cell configuration by using a 40 ms gap-free protocol at several negative holding-potentials (− 60 mV, − 80 mV and − 100 mV). All recordings were obtained with a D-6100 Darmstadt amplifier (List Medical, Germany), sampled at 10 kHz and filtered at 1 kHz. The pClamp8 or pClamp10.5 software (Molecular Devices, Sunnyvale, CA, USA) was used for pulse generation, data acquisition and subsequent analysis. By employing Fetchan and pStat softwares, the amplitude of single-channel currents was measured as the peak-to-peak distance in Gaussian fits of amplitude histograms. Channel activity (NP_O_, where N is the number of channels and P_o_ is the open probability of a single channel) at negative voltages was calculated by dividing the mean current amplitude of recordings lasting 40 s by the single-channel amplitude obtained from the same traces.

### Immunofluorescence

Transfected HEK293 cells were incubated with ConcanavalinA (ConA)-Rhodamine (Molecular Probes Ref. C860) for 20 min, rinsed with PBS and fixed with PFA 4%. For immunofluorescence staining cells were fixed 24 or 48 h post-transfection with paraformaldehyde 4% for 30 min. Upon thorough rinsing, cells were treated with a blocking solution of 5% bovine serum albumin (BSA) and 3% foetal bovine serum with or without triton 0.1% (for permeabilization). Anti-GFP antibody (Life Technologies, Cat # A-11122) was incubated overnight and Alexa-488 secondary antibody was used for detection. DAPI was used for nuclear staining.

Confocal imaging was performed to detect membrane expression of the GFP in the membrane (shown as ConA-Rhodamine staining) using the Leica SP5. Manders colocalization coefficient was calculated using home-made pipeline of CellProfiler (v.3.1.9)^62^. Only cells that expressed TRPP3-GFP above a threshold of intensity per unit area were considered. Pixel intensity profiles were obtained using ImageJ.

### Statistical analysis

Data are presented as the means ± S.E.M. Statistical tests included one-tail Student’s t-test, one-tail Mann–Whitney U-test, Kruskal–Wallis test followed by Dunn post hoc test, or one-way ANOVA followed by Bonferroni's post hoc test, as appropriate. Differences were considered significant if P < 0.05.

### Analysis of the 3D structure

The structures of PDB^63^ with code 5Z1W^18^, 5T4D^49^ and 5MKE^64^ were used to model the close conformation of TRPP3, and the structures with code 6DU8^19^, 6D1W^65^ and 5Z1W^18^ for the open state. Sequences were compared with ClustalOmega^66^, structural models were obtained with MODELLER^67^ and optimized with Rosetta. Superposition and visualization of structures were obtained with Matchmaker and Chimera^68^. Form with R278Q substitution was analysed by visual inspection of the neighbourhood of R278 after superimposing the structures 5Z1W and 6DU8. Conformations of the closed-state of wild type and R378W substitution forms were optimized with FIXBB and RELAX, from Rosetta package^69^. Several conformations of the linker between S2 and S3 were modelled for the open-state conformation (a total of 2500 structures). The loop between amino-acid 378 and 384 was remodelled without template to allow for the flexible changes caused upon substitution. The side-chains of the structures were further relaxed with 10 iteration cycles of FIXBB. We calculated the distribution of distances between the amino chemical groups of K452 and K455 and the oxygens Oδ1 and Oδ2 of the carboxyl group of D390, grouping them in hydrogen-bonding (at less than 3.5A) and non hydrogen-bonding (at more than 3.5A), where we use an extra margin of 0.5A to accept the hydrogen bond. We used Chimera for the visual inspection and analysis of selected structures with and without hydrogen bonds D390-K452 and D390-K455 of the open state conformations. We have to note that this is a study on static models, different than a molecular dynamics simulation of the protein embedded in a solvated environment. The study characterizes the main structural features of several models obtained by simulated annealing and optimization of the protein conformation.

### Sensory data and sour taste sensitivity test

Sensory data was obtained from a total of 44 participants (22 males and 22 females ranging from 15 to 57 years old). 13 participants were self-reported as carrying West African ancestry, 18 European ancestry and 13 Afroasiatic Ethiopian ancestry. In this study it was not possible to obtain Gumuz participants among the Ethiopians.

A modified Harris-Kalmus test^46^ was designed to determine the recognition threshold of sour taste of individuals. Citric acid (CA) or E-330 was used as stimuli since it is a harmless food additive which maximum doses are catalogued as “quantum satis” after food and health agencies. The citric acid used for this study was bought from Sigma-Aldrich with reference C0759.

The stimuli used were solutions of CA in Millipore-filtered, deionized water. They are prepared 24–48 h in advance of use, stored under refrigeration in amber glass bottles and warmed to room temperature before use. Water blanks are Millipore-filtered deionized water stored and handled the same way. The CA concentrations ranged from 5 × 10^–2^ to 1 mM in a 5 × 10^–2^ mM step.

As previously reported^70^, during each trial, subjects received a 10 mL sample in a medicine cup. After holding the sample in their mouth for at least 5 s the subjects attempted to identify the quality of taste (sweet, sour, bitter, salty, water). Subjects rinsed at least twice with deionized water. The threshold run begins with the lowest concentration. Subjects sampled each concentration once in ascending order until they identify the target quality “sour”. We next ensure that the subjects experienced a reliable taste sensation with a sorting task. Subjects receive 3 samples at the concentration at which they identified the target quality with 3 more blank samples. All 6 samples are presented at the same time in a random position. The subjects are asked to sort 3 cups into “tastes” and 3 cups into “waters”. If the subject can correctly sort the 6 samples in 2 consecutive trials, the threshold run ends. If a subject fails to sort correctly the sorting task is repeated at the next higher concentration. The concentration that first allows successive correct sorts is the taste quality recognition threshold.

### Saliva sample collection, DNA extraction, genotyping and allele calling

After having written informed consent from the participants and/or their parents, we obtained a saliva sample from which DNA was extracted using NaCl 6 M solution and subsequently precipitating the DNA on isopropanol^71^. Genotyping was performed through Sanger sequencing of PCR fragments. The primers used for the PCR are the following:

**Table:** 

Target	Forward primer	Reverse primer
rs17112895 and rs7909153	5′-AAGATGACCACCAGGTCCAG-3′	5′-TTGATACGTGGAGCAGCAAG-3′
rs7909153	5′-GTCAGAAGGAGGGCTTAGGG-3′	5′-CTGATCCGCTATGTCAGCAA-3′
rs17112895	5′- GCACCTCCTGTAGCTGGAAA-3′	5′- CATTGCCTTGGGAGATGAAC-3′

The primers used for sequencing are:

**Table:** 

	Sequencing Primers
Seq1	Reverse 5′-ACTGAAACTCAAATGTAAATCTG-3′
Seq2	Forward 5′-CATGCACAAGAGACGGGAAC-3′
Seq3	Reverse 5′-CATTGCCTTGGGAGATGAAC-3′

The PCR reactions were sent to Eurofins to perform Sanger sequencing. The program 4Peaks was used to visualize the electropherograms and manually validate that the variants were appropriately called for each sample.

### Ethical approval

The study of sour test sensitivity, with reference number 2018/8294/I, was approved by the ethical committee CEIm-Parc de Salut MAR and written informed consent to participate in the study and for the saliva donation was obtained from all participants and/or their parents. All methods to obtain samples and data were performed in accordance with the relevant guidelines and regulations, including the Helsinki Declaration of 1964, as revised in October 2013 (Fortaleza, Brazil).

## Supplementary information

Supplementary Information 1.

Supplementary Information 2.

Supplementary Information 3.

Supplementary Information 4.

Supplementary Information 5.

## Data Availability

The authors declare that the data supporting the findings of this study are available within the article and its Supplementary Information file, or are available from the authors upon request.
