# Supplementary material for: Adaptive selection drives TRPP3 loss-of-function in an Ethiopian population
Source: Sci Rep. 2020 Dec 2;10:20999. doi: 10.1038/s41598-020-78081-z (PMC7710729; doi:10.1038/s41598-020-78081-z)
Supplement: Supplementary file 1 — Supplementary Information 1. [file 41598_2020_78081_MOESM1_ESM.pdf]

SUPPLEMENTARY INFORMATION TO:

ADAPTIVE SELECTION DRIVES TRPP3 LOSS-OF-FUNCTION IN AN ETHIOPIAN POPULATION

Sandra Walsh[1], Mercè Izquierdo-Serra[2], Sandra Acosta[1], Albert Edo[2], Maria Lloret[2], Roser Moret[1], Elena Bosch[1], Baldo Oliva[3], Jaume Bertranpetit[1*,] José Manuel Fernández-Fernández[2*]

1. Institut de Biologia Evolutiva (UPF-CSIC), Universitat Pompeu Fabra, Dr. Aiguader, 88, 08003 Barcelona, Catalonia, Spain.

2. Laboratory of Molecular Physiology, Department of Experimental and Health Sciences, Universitat Pompeu Fabra, Barcelona Spain

3. Structural Bioinformatics Lab, Department of Experimental and Health Science, Universitat Pompeu Fabra, Barcelona, Spain.

*co-corresponding authors: jaume.bertranpetit@upf.edu and jmanuel.fernandez@upf.edu

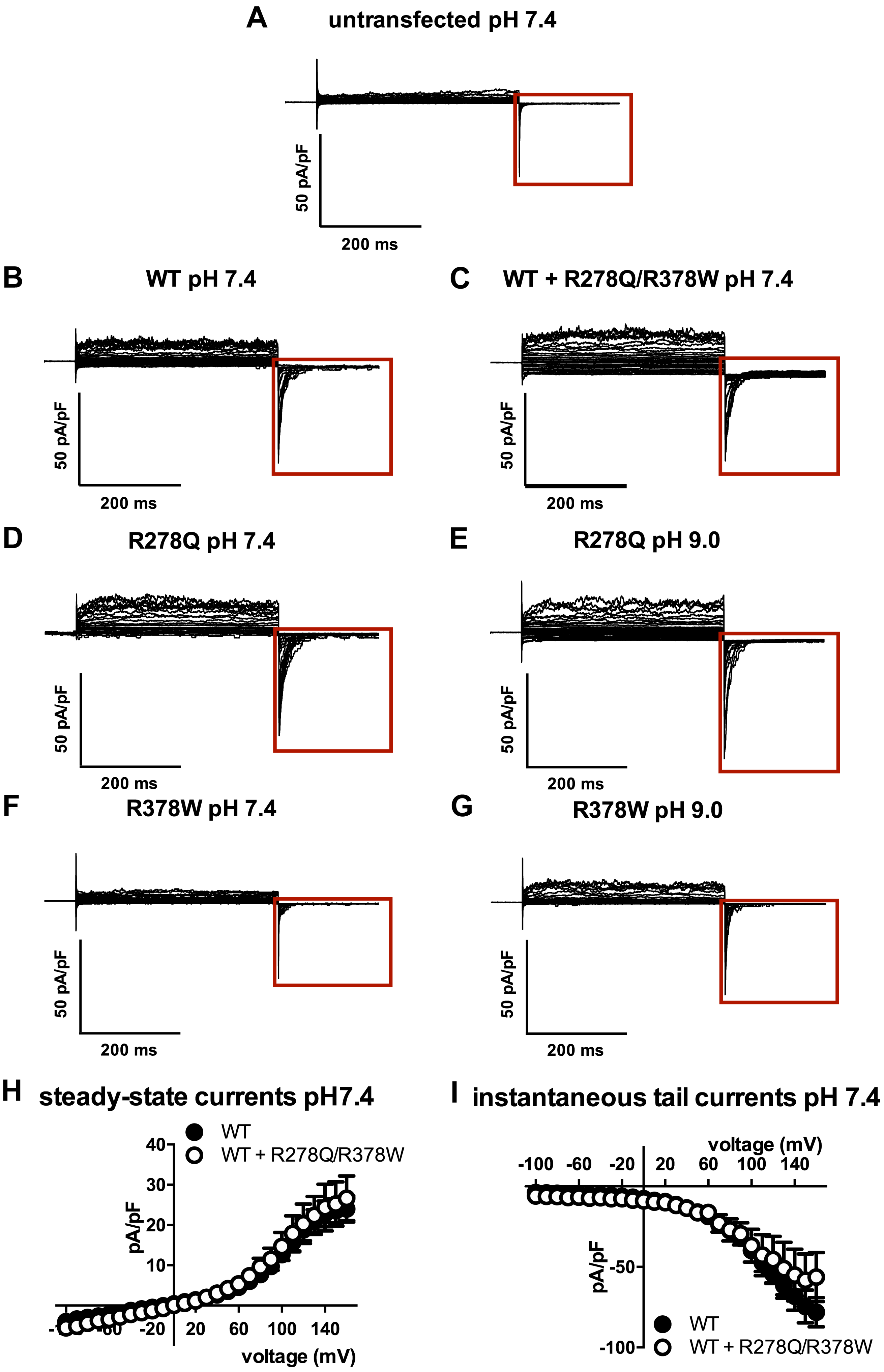
A
untransfected pH 7.4
50 pA/pF
200 ms
B
WT pH 7.4
50 pA/pF
200 ms
C
WT + R278Q/R378W pH 7.4
50 pA/pF
200 ms
D
R278Q pH 7.4
50 pA/pF
200 ms
E
R278Q pH 9.0
50 pA/pF
200 ms
F
R378W pH 7.4
50 pA/pF
200 ms
G
R378W pH 9.0
50 pA/pF
200 ms
H
steady-state currents pH7.4
WT
WT + R278Q/R378W
40
30
20
10
pA/pF
-20
20
60
100
140
voltage (mV)
I
instantaneous tail currents pH 7.4
voltage (mV)
-100
-60
-20
20
60
100
140
-50
-100
pA/pF
WT
WT + R278Q/R378W

Supplementary Figure S1. Illustrative whole-cell recordings for the loss-of-function effects of single mutations R278Q and R378W, and lack of dominant negative action of double mutant R278Q/R378W.

(**A**-**G**) Representative whole-cell currents from untransfected HEK293 cells or HEK 293 cells co-transfected with cDNAs encoding PKD1L3 and wild-type (WT), R278Q, R378W or WT+R278Q/R378W TRPP3-GFP, and exposed to either extracellular pH 7.4 or pH 9.0, as indicated. Currents were elicited by step pulses from -100 to +160 mV in 10 mV increments with a postpulse to -100 mV, and normalized by cell size (membrane capacitance). Red boxes show tail currents after membrane repolarization to -100 mV. (**H**-**I**) Average current-voltage (I-V) relationships for steady-state currents (**H**) and instantaneous tail currents (**I**) at each test pulse in HEK293 cells expressing PKD1L3 and wild-type (WT) or WT+double mutant R278Q/R378W TRPP3-GFP and exposed to extracellular pH 7.4. Area under the I-V curves (AUCs) were calculated for statistical analysis (only values at positive voltages were taken under consideration for steady-state I-V curves). Obtained AUC values for steady-state currents were: WT (●, n=19) 1662.6±233.9; WT+R278Q/R378W (○, n=12) 1829.3±395 (P=0.44, one-tail Mann-Whitney U-test). Obtained AUC values for instantaneous tail currents were: WT (●, n=19) -6034.2±649.3; WT+R278Q/R378W (○, n=12) -5510.4±1285.4 (P=0.15, one-tail Mann-Whitney U-test).

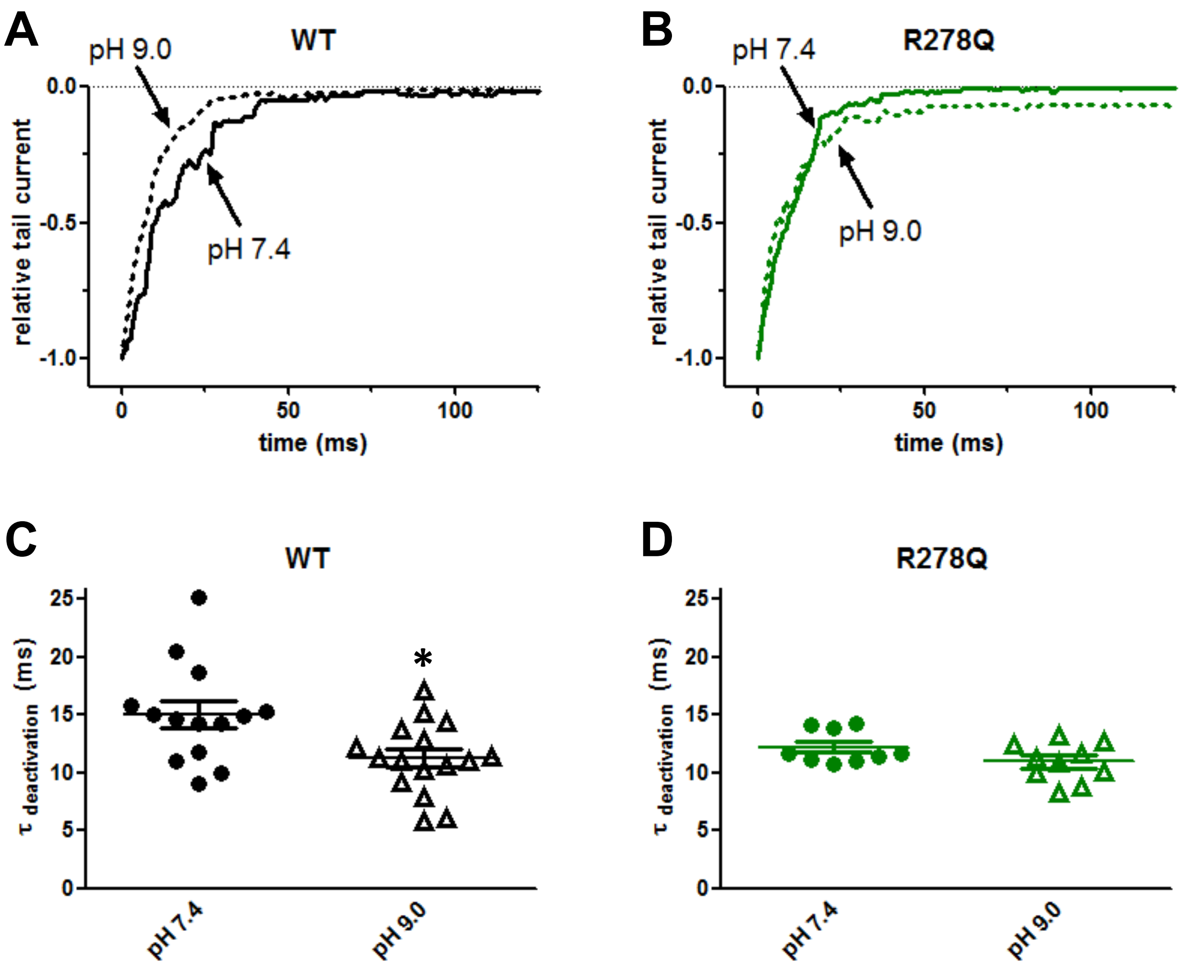

Supplementary Figure S2. Deactivation kinetics of WT and R278Q TRPP3 channels at both neutral (pH 7.4) and alkaline (pH 9) conditions.

(**A**, **B**) Representative deactivation of tail currents after depolarization to +160 mV and subsequent repolarization to -100 mV from HEK293 cells co-transfected with cDNAs encoding PKD1L3 and wild-type (WT) or R278Q TRPP3-GFP, and exposed to either extracellular pH 7.4 or pH 9.0, as indicated. The tail current was normalized to each peak amplitude. (**C**, **D**) Average time constant of deactivation of tail currents recorded at the above mentioned experimental conditions. *P<0.05 (Kruskal-Wallis , followed by Dunn post hoc test).

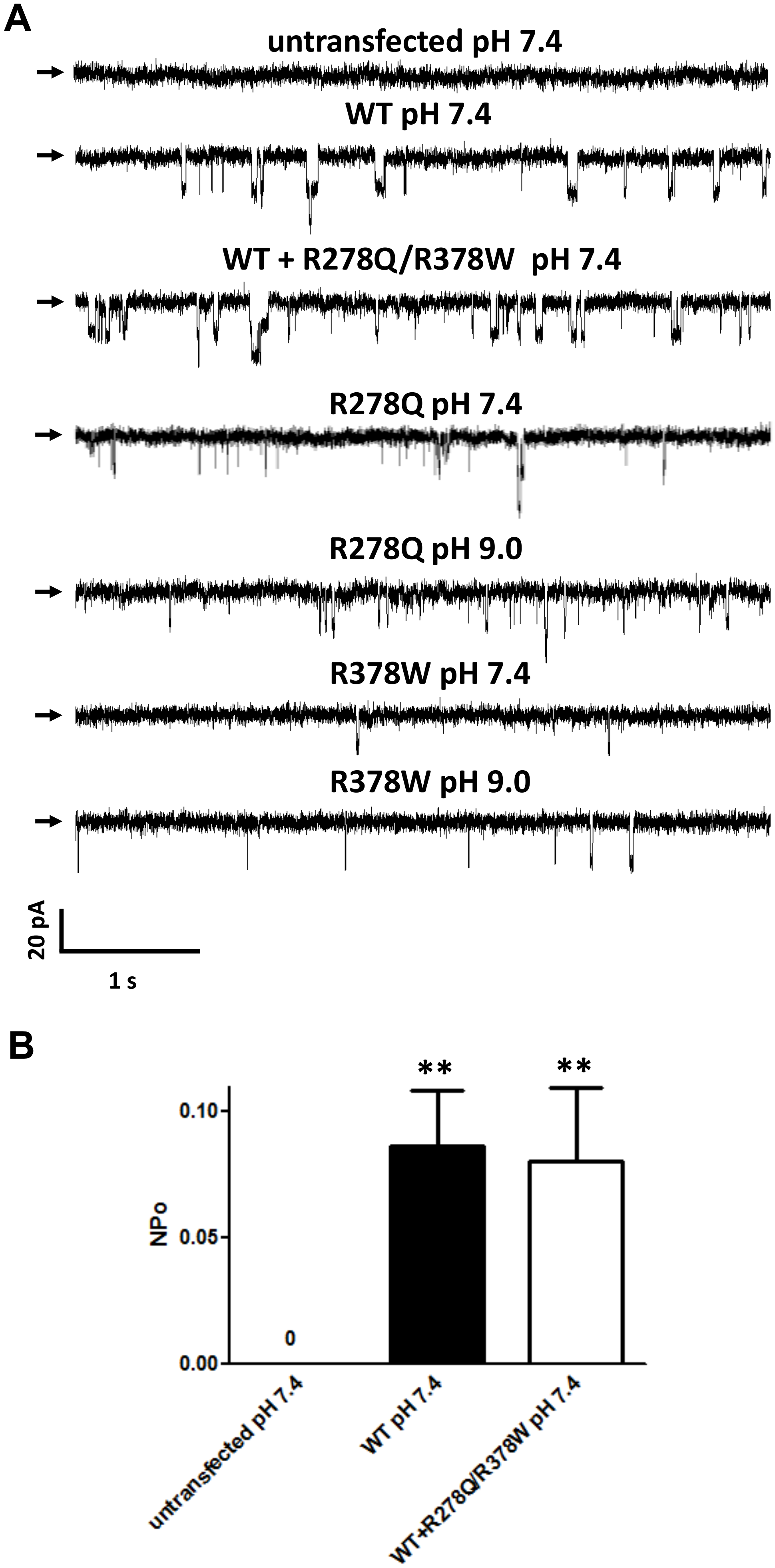
A
untransfected pH 7.4
WT pH 7.4
WT + R278Q/R378W pH 7.4
R278Q pH 7.4
R278Q pH 9.0
R378W pH 7.4
R378W pH 9.0
20 pA
1 s
B
**
**
0.10
0.05
0.00
NPo
0
untransfected pH 7.4
WT pH 7.4
WT+R278Q/R378W pH 7.4

Supplementary Figure S3. Illustrative recordings for the action of single mutations R278Q and R378W on spontaneous TRPP3 activity, and lack of dominant negative effect of the double mutant R278Q/R378W channel.

(**A**) Representative single-channel activity of the TRPP3 single mutants in whole-cell recordings obtained at negative membrane potential (-80 mV) from untransfected HEK293 cells or HEK 293 cells co-transfected with cDNAs encoding PKD1L3 and wild-type (WT), R278Q, R378W or WT+R278Q/R378W TRPP3-GFP, and exposed to either extracellular pH 7.4 or pH 9.0, as indicated. Arrows indicate the zero current level. (**B**). Average channel activity (NP_O) in whole-cell recordings obtained at -80 mV and pH 7.4 from untransfected HEK293 cells (n=6) and HEK 293 cells co-transfected with cDNAs encoding PKD1L3 and WT (n=16) or WT+R278Q/R378W (n=7) TRPP3-GFP. **P<0.01 (when compared to the control untransfected condition; Kruskal-Wallis, followed by Dunn`s Multiple Comparisons post hoc test).

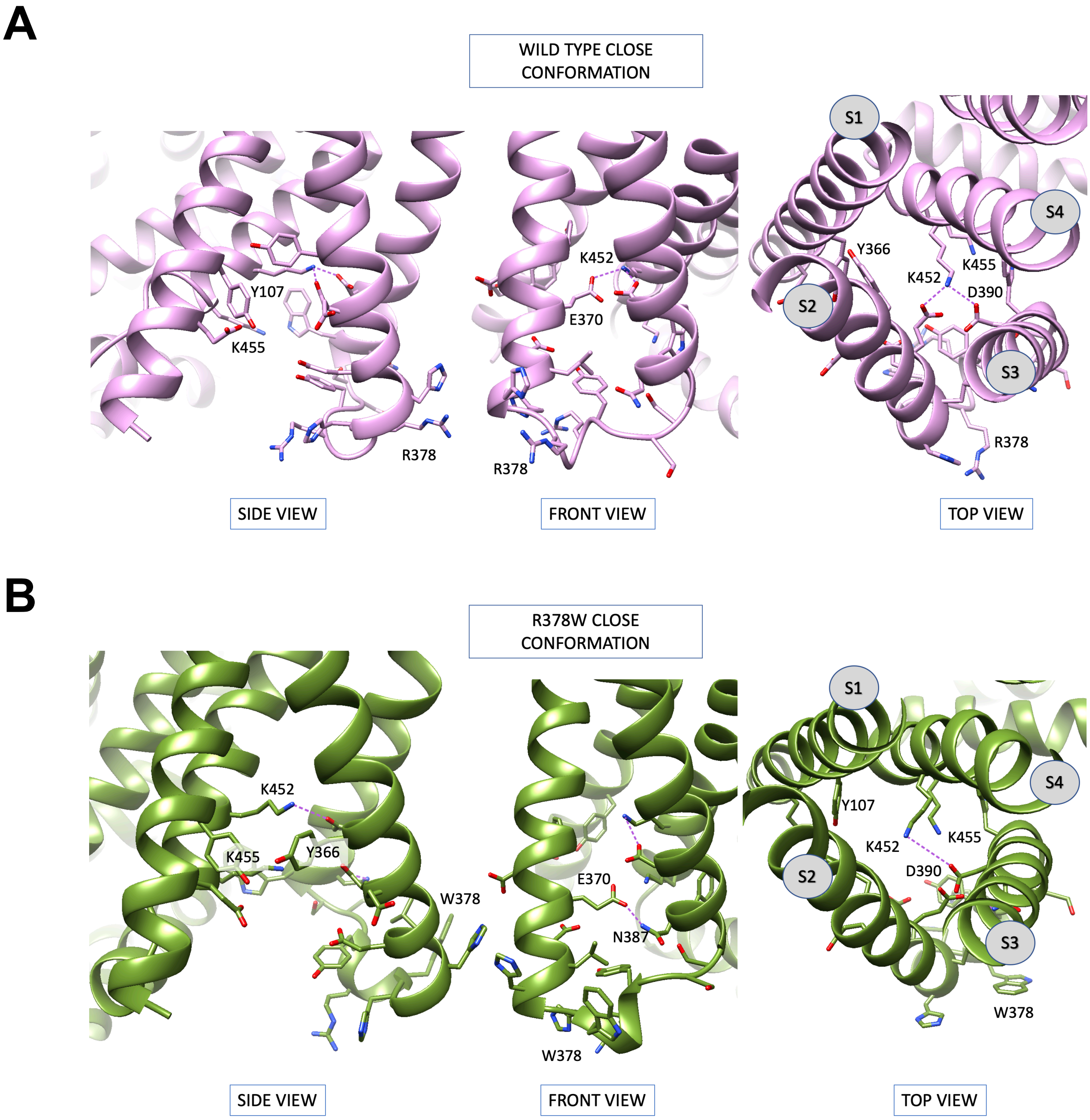

Supplementary Figure S4. Ribbon plot of the close-state conformation of wild-type and R378W mutant forms.

(**A**) Frontal, lateral-side and top views of the closed-state conformation of wild-type form showing the hydrogen-bond between D390 and K452 and the orientation of most relevant side-chains: amino-acids in the linker between S2 and S3 (this includes R378) and K455, Y107, Y366, N387, E370 and E373, that are involved in the interactions between S2, S3 and S4 helices. (**B**) Frontal, lateral-side and top views of the closed-state conformation of R378W mutant form, showing the same amino-acids and relevant hydrogen bonds as in (A) and the reorientation of the side-chain of tryptophan in 378 that stabilizes the linker helix between S2 and S3. Helices S1, S2, S3 and S4 are also indicated in the top view of figures A and B.

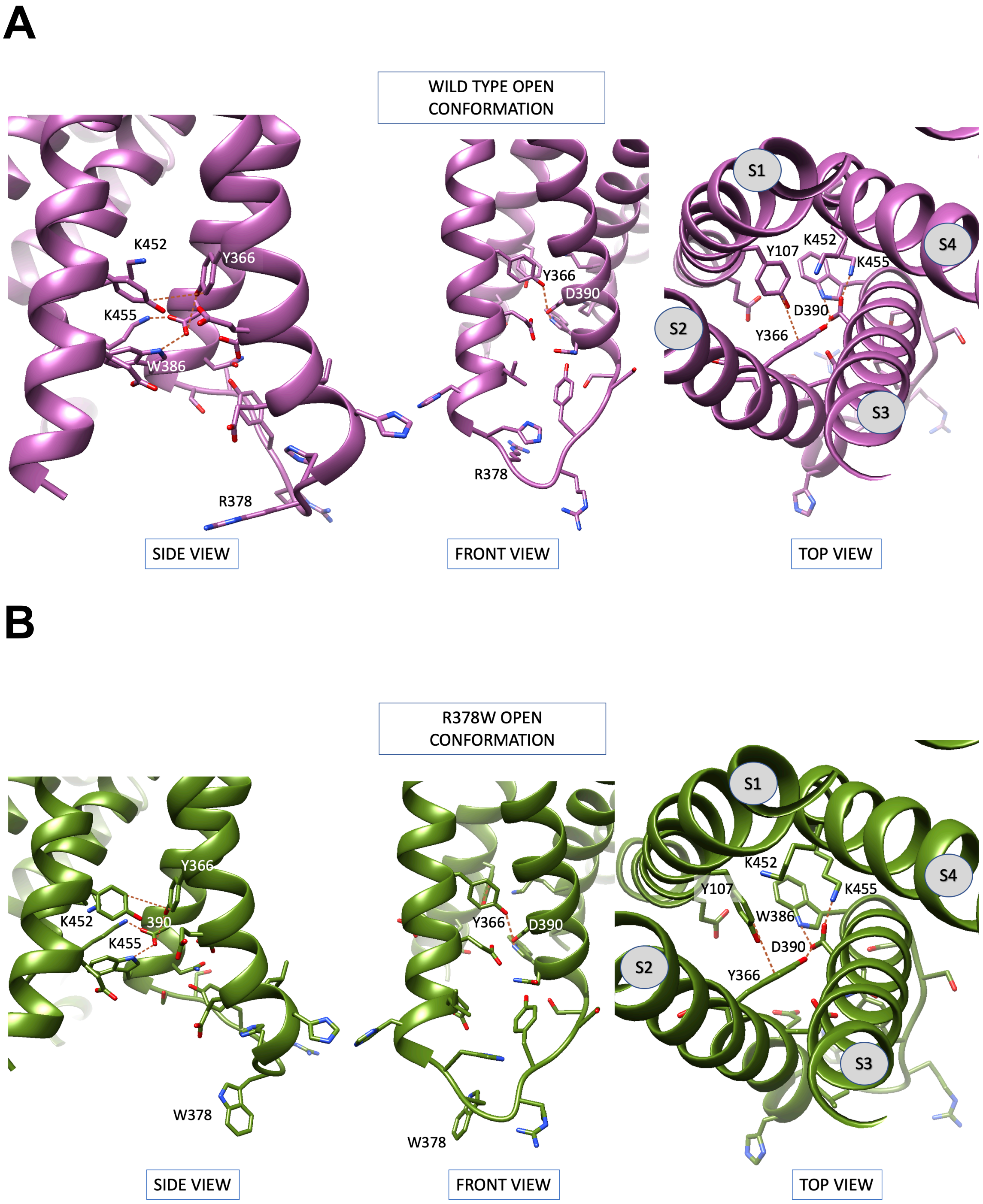

Supplementary Figure S5. Ribbon plot of selected models in open-state conformation of wild-type and R378W mutant forms.

(**A**) Frontal, lateral-side and top views of the open-state conformation of wild-type form showing the lost hydrogen-bond between D390 and K452, the gain of D390-K455 and the orientation of most relevant side-chains: amino-acids in the linker between S2 and S3 (this includes R378) and Y107, Y366, N387, E370 and E373, that are involved in the interactions between S2, S3 and S4 helices. (**B**) Frontal, lateral-side and top views of the open-state conformation of R378W mutant form, showing the same amino-acids and relevant hydrogen bonds as in (A), while the side-chain of W378 is oriented after optimization as the native arginine. Helices S1, S2, S3 and S4 are also indicated in the top view of figures A and B.

A

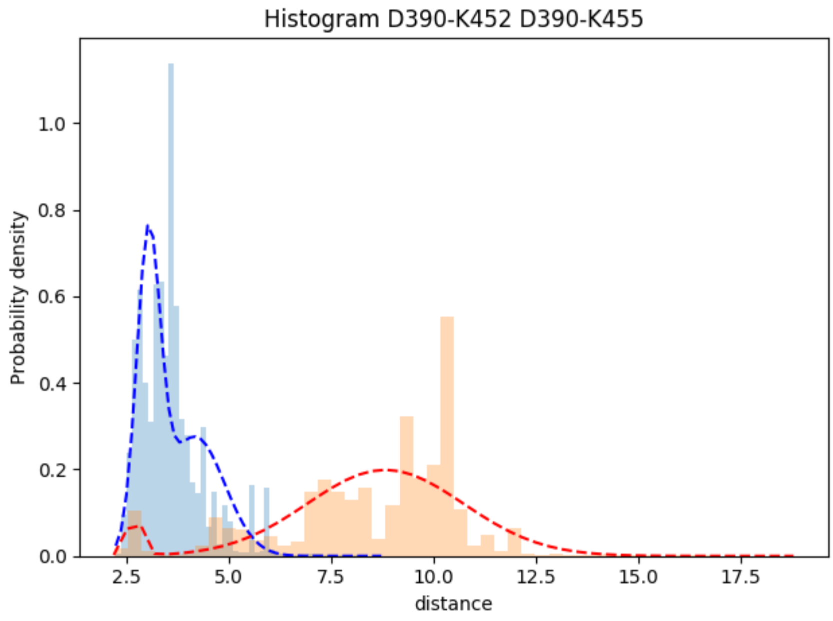

B

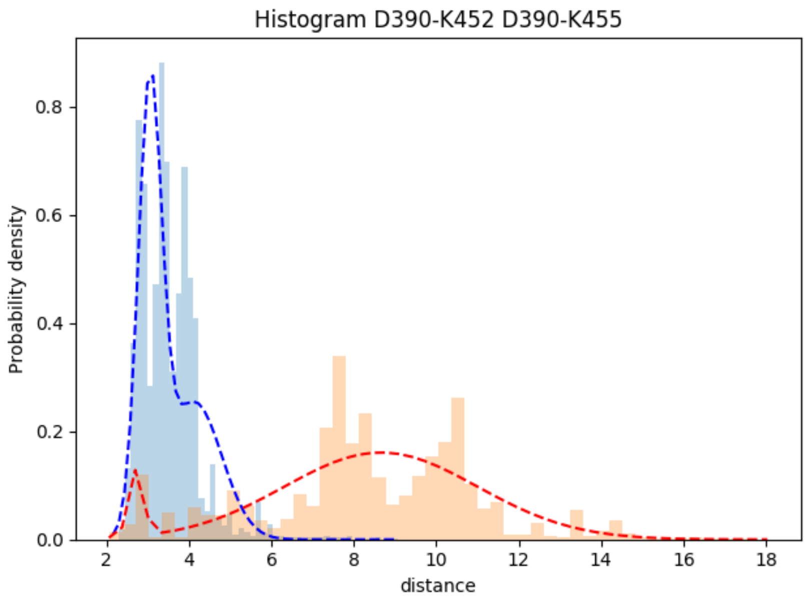

Supplementary Figure S6. Distribution of contacts between D390 and lysines K452 and K455.

(**A**) Histogram of the contacts of the models of the wild type form. (**B**) Histogram of the contacts of the models of the R378W mutant form. The distribution of the closest distance between the carboxyl group of D390 and the amino group of K452 is shown in cyan, while the distribution with K455 is in orange. A curve is fitted for each distribution (blue for D390-K452 and red for D390-K455 distances) with a bimodal formed by the sum of two gaussian approximations: one with the contacts at H-bond distance (shorter than 3.5Å) and the other with the rest.

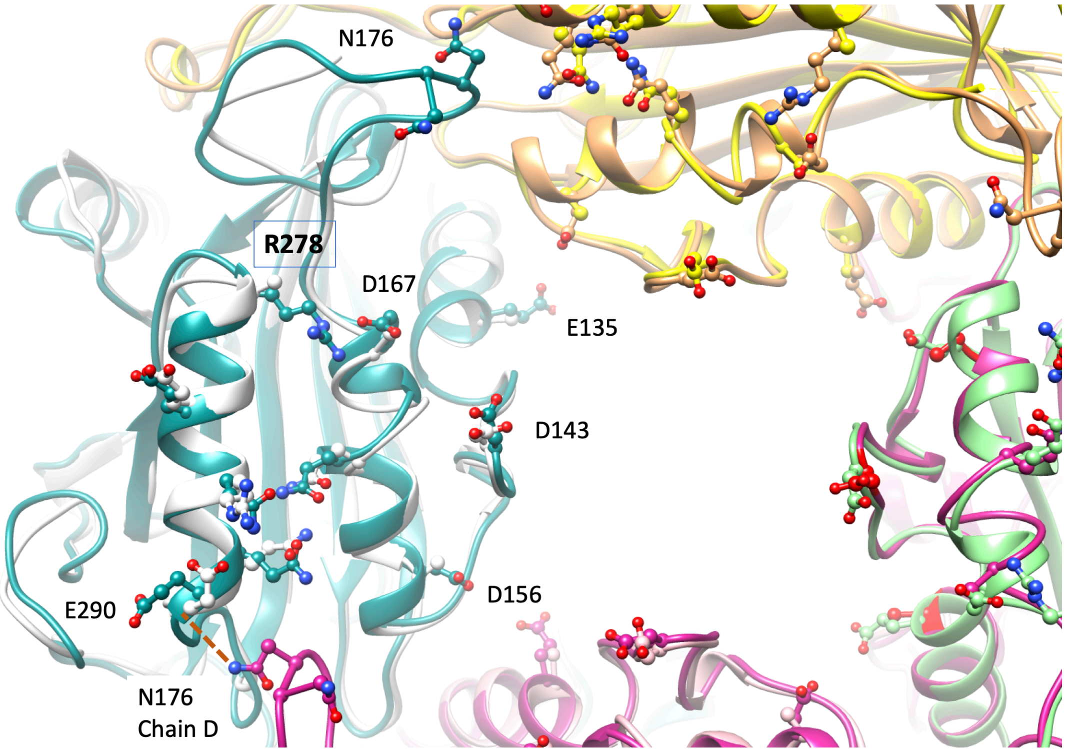

Supplementary Figure S7. Ribbon plot highlighting the position of R278 and its environment.

Representation of the superposition of structures of chain B of 6DU8 (white) and chain D of 5Z1W (blue). Side chains of relevant amino-acids are shown in ball and sticks: D143, D156, E135, D167, E290, N176. The hydrogen bond between the peptide bond of E290 and the side-chain of N176 from the closest partner (chain B of 5Z1W in magenta) is also shown.

	Population	Sample	Sex	Age	pH	Recognition (mM)	rs17112895	R278Q	rs7909153	R378W
	WA	S1	F	57	-	0.3	CGA/CGA	R/R	CGG/CGG	R/R
	WA	S2	F	53	-	0.15	CGA/CGA	R/R	CGG/CGG	R/R
	WA	S3	F	20	-	0.15	CGA/CGA	R/R	CGG/CGG	R/R
	WA	S4	M	19	-	0.15	CGA/CGA	R/R	CGG/CGG	R/R
	WA	S5	F	32	-	0.25	CGA/CGA	R/R	CGG/CGG	R/R
	WA	S6	M	35	-	0.3	CGA/CGA	R/R	CGG/CGG	R/R
*	WA	S7	M	29	-	0.35	CAA/CGA	Q/R	TGG/CGG	W/R
	WA	S8	M	23	-	0.35	CGA/CGA	R/R	CGG/CGG	R/R
*	WA	S9	M	28	-	0.25	CAA/CGA	Q/R	TGG/CGG	W/R
	WA	S10	F	34	-	0.15	CGA/CGA	R/R	CGG/CGG	R/R
	WA	S11	M	24	-	0.3	CGA/CGA	R/R	CGG/CGG	R/R
*	WA	S12	M	41	-	0.25	CAA/CGA	Q/R	TGG/CGG	W/R
	EU	S13	F	31	-	0.25	-	-	-	-
	EU	S14	M	25	7	0.35	-	-	-	-
	EU	S15	M	28	8	0.3	CGA/CGA	R/R	CGG/CGG	R/R
	EU	S16	F	31	6.25	0.3	CGA/CGA	R/R	CGG/CGG	R/R
	EU	S17	M	28	6.5	0.35	CGA/CGA	R/R	CGG/CGG	R/R
	EU	S18	M	28	7	0.25	CGA/CGA	R/R	CGG/CGG	R/R
	EU	S19	F	37	6.75	0.15	CGA/CGA	R/R	CGG/CGG	R/R
	EU	S20	F	45	6	0.25	CGA/CGA	R/R	CGG/CGG	R/R
	EU	S21	F	22	6.25	0.2	CGA/CGA	R/R	CGG/CGG	R/R
	EU	S22	F	28	6.75	0.2	CGA/CGA	R/R	CGG/CGG	R/R
	EU	S23	F	22	6	0.25	-	-	CGG/CGG	R/R
*	ET	S24	M	24	7	0.2	CAA/CAA	Q/Q	TGG/TGG	W/W
	ET	S25	F	22	8.5	0.15	CGA/CGA	R/R	CGG/CGG	R/R
	ET	S26	F	22	6.5	0.2	CGA/CGA	R/R	CGG/CGG	R/R
*	ET	S27	M	49	5.5	0.2	CAA/CGA	Q/R	TGG/CGG	W/R
	ET	S28	M	23	8	NA	CGA/CGA	R/R	CGG/CGG	R/R
	EU	S29	F	22	5	0.1	CGA/CGA	R/R	CGG/CGG	R/R
	EU	S30	F	22	7	0.25	CGA/CGA	R/R	CGG/CGG	R/R
*	WA	S31	F	23	7	0.15	CAA/CGA	Q/R	TGG/CGG	W/R
	EU	S32	F	22	7	0.15	CGA/CGA	R/R	CGG/CGG	R/R
	EU	S33	F	22	7	0.2	CGA/CGA	R/R	CGG/CGG	R/R
	EU	S34	M	22	6	0.25	CGA/CGA	R/R	CGG/CGG	R/R
	EU	S35	M	22	8.25	0.45	CGA/CGA	R/R	CGG/CGG	R/R
	EU	S36	F	22	8	0.25	CGA/CGA	R/R	CGG/CGG	R/R
	ET	S37	M	24	6.75	0.3	CGA/CGA	R/R	CGG/CGG	R/R
	ET	S38	M	32	6.5	0.2	CGA/CGA	R/R	CGG/CGG	R/R
	ET	S39	M	33	8	0.45	CGA/CGA	R/R	CGG/CGG	R/R
	ET	S40	M	15	7	0.2	CGA/CGA	R/R	-	-
	ET	S41	M	17	7.5	0.25	CGA/CGA	R/R	CGG/CGG	R/R
	ET	S42	F	15	6	0.2	CGA/CGA	R/R	CGG/CGG	R/R
	ET	S43	M	19	6	0.1	CGA/CGA	R/R	CGG/CGG	R/R
	ET	S44	F	19	6.5	0.25	CGA/CGA	R/R	CGG/CGG	R/R

Supplementary Table S1. Detailed summary of the sampled individual's genotypes and sour taste phenotypic analysis. The table reports individuals' ancestry, Ethiopian (ET), West African (WA) or European (EU), sample number, sour recognition threshold (CA mM), saliva pH and genotype for both variants. Individuals carrying derived alleles are marked with an asterisk (*).
